# Chronic disease related emergency department presentations and potential for redirection to alternative acute care settings (“FOCUS” study): A nationwide flashmob study

**DOI:** 10.1371/journal.pone.0353157

**Published:** 2026-07-15

**Authors:** Elisabeth M. Mols, Michelle N. Körnmann, Harm R. Haak, Jelmer Alsma, Patricia M. Stassen, Karin A. H. Kaasjager, Geert H. Groeneveld, Marjolein N. T. Kremers, on behalf of the ORCA (Onderzoeks Consortium Acute Geneeskunde) Acute Medicine Research Consortium and Local Investigators

**Affiliations:** 1 Department of Internal Medicine, Máxima MC, Veldhoven/Eindhoven, The Netherlands; 2 Department of Health Services Research and CAPHRI School of Public Health and Primary Care, Ageing and Long Term Care, Maastricht University, Maastricht, The Netherlands; 3 Department of Internal Medicine, Section Of Acute and General Internal Medicine, Amsterdam University Medical Center, Location VU, Amsterdam, The Netherlands; 4 Amsterdam Public Health, Quality of Care, Global Health, Amsterdam, The Netherlands; 5 Department of Internal Medicine, Section Acute Medicine, Erasmus University Medical Center, Rotterdam, The Netherlands; 6 Department of Internal Medicine, Maastricht University Medical Centre+, Maastricht, The Netherlands; 7 Department of Acute Internal Medicine, University Medical Center Utrecht, Utrecht, The Netherlands; 8 Department of Internal Medicine, Section of Acute Internal Medicine, Leiden University Medical Center, Leiden, The Netherlands; 9 Department of Internal Medicine, Catharina Ziekenhuis Eindhoven, Eindhoven, The Netherlands; Adelaide University, AUSTRALIA

## Abstract

**Background:**

Emergency Departments (EDs) face increasing pressure, driven in part by the complex needs of an ageing population. Many internal medicine ED visits may reflect complications of chronic conditions. Aiming to maintain the accessibility of acute care, evaluation whether such presentations can be managed in alternative settings is necessary.

**Methods:**

We conducted a prospective multicentre flashmob study in 28 Dutch hospitals over a 24-hour period (November 28^th^ and 29^th^, 2024). All adult non-elective ED patients presenting for internal medicine were included. Primary outcomes were the proportion of visits related to chronic conditions and the potential for redirection to an alternative acute care facility. Data on patient characteristics, case complexity, and organisational factors were collected and analysed using descriptive and comparative statistics.

**Results:**

Among 203 included patients, 45.3% presented with an acute complication of a chronic condition. Of these patients, 39.4% were receiving chronic care from an internist. Nearly one-quarter (24.1%) of all ED visits were considered preventable. Additionally, 39.9% of patients could have been managed in alternative settings such as acute outpatient clinics or directly to acute admission units. Despite the presence of these alternative care facilities in most hospitals, the lack of structural organisation was reported in 58.0% as a barrier to redirecting patients.

**Conclusion:**

A substantial proportion of internal medicine patients at the ED reflect an acute on chronic care need. While alternative care pathways exist, organisational barriers hinder their use. Improving access to structured outpatient acute care could possibly reduce ED use and improve the efficiency of acute internal medicine services.

## Introduction

The pressure on acute care is increasing, as illustrated by the rising number of overcrowded Emergency Departments (EDs) and ED diversions [1,2]. ED crowding is widely recognised as a complex, system-level problem resulting from a mismatch between demand and available capacity in the acute care chain [2,3]. Demographic changes, with an ageing population and a higher prevalence of multimorbidity, are often cited as one explanation, as they contribute to increasing care complexity and demand [2–5].These patients often require comprehensive diagnostics and hospital admission, resulting in prolonged ED length of stay (LOS) and downstream capacity constraints, which in turn exacerbates crowding [6].

Over recent decades, EDs have transformed into highly specialised environments designed to deliver rapid, high-intensity care for critically ill patients in need for specialised interventions. However, a shift in the acute care landscape has occurred, with an increasing number of patients presenting to the ED due to deterioration or complications of pre-existing chronic illnesses [7–9]. This group often represents a complex and vulnerable population, with higher rates of comorbidities, polypharmacy, and frailty [7]. For these patients, continuity of care within their chronic care network is essential to initiate timely and appropriate management. Moreover, a proportion of these patients presents with subacute care needs that may not require the full specialised capacity of the ED.

Given the ageing population, the evolving care demands and increasing case-complexity, ensuring the provision of the right care in the right setting is essential for maintaining a sustainable and future-proof acute care system. Achieving this requires insight into the type of patients presenting to the ED. The Dutch Society of Internal Medicine (NIV) has suggested that a substantial proportion of internal medicine ED visits are related to deterioration of chronic conditions, summarised in the phrase “Acute care is Chronic care” [10]. However, the exact proportion of such visits and the potential for redirection remains unclear. Addressing this knowledge gap is important, as pro-active management could prevent these acute presentations, supporting continuity of care and avoiding unnecessary ED use. For example, patients might be seen in alternative acute care settings, instead of the ED. In these settings, care could be provided by their own physician or by an acute internist, a specialist trained to manage complex acute internal medicine cases. Such redirection not only aligns with the principle of providing the right care in the right place, but is also increasingly important in the context of workforce shortages and ED overcrowding.

We conducted the FOCUS (Finding Optimal Care in Urgent Settings) study, a national multicentre flashmob-study, in which all patients presenting to the ED for internal medicine were analysed. The aim of this study was to quantify the proportion of ED visits related to chronic care trajectories and to assess the potential for redirection to alternative acute care settings, providing a foundation for optimising the delivery of care in the right place at the right time.

## Methods

### Study design

This study is an observational, cross-sectional, multicentre flashmob study conducted in the EDs of 28 Dutch hospitals, capturing a 24-hour snapshot of ED presentations. The study was conducted over a 24-hour period, from 08:00 on the 28th of November 2024 to08:00 on the 29th of November 2024. The flashmob method is a new way of conducting prospective research whereby relatively simple, but clinically relevant, questions can be answered in a short period of time [11]. Ethical approval was obtained from the Medical Ethics Review Committee (METC) of Máxima Medical Center (N24.040). Written informed consent was obtained before inclusion in the study.

### Study setting

In the Netherlands, emergency care is provided in 79 EDs, of which 76 operate 24/7. General practitioners (GPs) have a strong gatekeeping role, and referral is very common for access to the ED [12]. Dutch EDs function as an open format department, providing acute care for all medical specialties. Upon presentation or after clinical assessment, patients are registered under a specific specialty (e.g., internal medicine) but may be assessed by an emergency physician (EP) or by a clinician from the corresponding specialty.

### Study population

The study included all patients ≥18 years presenting to the ED for internal medicine (i.e., referred to internal medicine, triaged to internal medicine, or for whom the internist ultimately acted as the lead treating physician), encompassing all subspecialties, including endocrinology, haematology, infectious diseases, nephrology, oncology, rheumatology, vascular, allergy and immunology, and geriatric medicine, at participating hospitals during the study period. All eligible patients presenting during the study period were consecutively included, to minimise the risk of selection bias. To ensure a representative sample of hospital care, the study included patients from university, teaching, and general hospitals.

### Study outcomes

The primary outcomes of this study were:

1. The proportion of patients presenting with an acute complication, exacerbation or deterioration related to a chronic illness. For the purpose of this study, ‘complication’ refers to any complication, exacerbation or deterioration of a chronic condition, as assessed by the primary responsible physician.
2. The proportion of patients under treatment with internal medicine, the GP, or other medical specialties, defined as those with at least one registered medical encounter within the preceding year.
3. The proportion of ED visits that could have been redirected elsewhere in the emergency care chain or prevented, as retrospectively assessed by the primary responsible physician.

Secondary outcomes included organisational characteristics of acute care delivery, case complexity of patients presenting to the ED for internal medicine (determined by age, comorbidities, polypharmacy, and frailty), and the proportion of patients who had contact with the hospital within seven days prior to ED visit.

### Data collection

Data were collected using Castor EDC. Before deployment, the electronic Case Report Form (eCRF) underwent pilot testing with practicing internists to ensure that all items were comprehensible and interpreted as intended. The eCRF consisted of two main components. First, a one-time organisational questionnaire was completed by the local investigators of all participating hospitals before the start of the study (S1 Questionnaire).

Second, patient level data were collected by the primary responsible physician at ED disposition through a separate eCRF, consisting of two sections (S2 Questionnaire). The first section captured clinical and demographic characteristics. The second section captured the primary responsible physicians assessment of the chronic disease related aspects of the presentation and the potential for alternative management. The primary responsible physician was asked to answer three key questions:

1. Is the patient presenting with an acute deterioration of a chronic disease, a complication of the treatment of a chronic disease, or a complication related to a chronic disease?
2. Do you think that the patient’s presentation to the ED, given the current organisation of care, could have been prevented?
3. Could the patient, in theory, have been managed in a setting other than the ED for this complaint?

### Definitions and variables of interest

Details on variables collected for organisational and patient characteristics are presented in Table 1.

**Table 1:** Definitions of variables.

Organisational characteristics	
Hospital type	University, Teaching, General
Internal medicine beds	Theoretical number of beds (including subspecialties: acute medicine, infectious diseases, geriatrics, vascular medicine, allergology, endocrinology, hematology, nephrology, and oncology) in the hospital, irrespective of temporary closures (e.g., due to staffing shortages).
Acute care facility	Presence of a dedicated acute medical unit, short‑stay unit or observation unit, and the maximum length of stay (LOS).
Alternative acute care facility	Defined as facilities where patients with an acute care need can be assessed outside the ED (e.g., outpatient clinics, daycare units, inpatient wards).
**Patient characteristics**	
Demographics	Age, Sex
Urgency category	Urgency ED levels were determined according to the NTS (Dutch Triage Standard) or MTS (Manchester Triage Standard): U0 (red) reanimation, U1 (orange) life threatening, U2 (yellow) highly urgent, U3 (green) urgent, U4-U5 (blue) non-urgent.
Number of medications	Defined as prescribed medications (daily/weekly), drops, inhaled meds, and regular over-the-counter use (e.g., paracetamol).
Comorbidities	Comorbidities were defined as the presence of one or more chronic medical conditions co‑existing with the index condition at the time of the ED visit, categorised as No, 1–5 or >5
Frailty	Frailty was defined according to the Clinical Frailty Scale (CFS): 1–3 were combined as “fit”, 4–6 as “vulnerable to moderately frail” and 7–9 as “severely frail to terminally ill”.
Prior hospital contact	Any hospital contact in the 7 days prior to the ED visit, including telephone contact, outpatient visits, ED visits or inpatient admissions.

### Statistical analysis

We summarised baseline patient and hospital characteristics using descriptive statistics. Differences between patients with chronic and non-chronic care trajectories, complexity measures, and other outcomes were tested with Pearson’s chi-squared or Fisher’s exact tests for categorical variables, and independent samples t-tests or Mann-Whitney U tests for continuous variables, as appropriate. Hospital-level differences in acute care organisation were analysed using the same approach. A p-value of <0.05 was considered statistically significant. Analyses were performed in SPSS version 25 (IBM Corp, Armonk, NY, USA).

## Results

### Organisational characteristics

A total of 28 hospitals participated in the study, comprising 6 university hospitals, 14 teaching hospitals, and 8 general hospitals (Table 2). All participating hospitals had an ED operating 24/7. A total of 24 hospitals had an acute care facility for patient admissions, the majority of which were acute medical units (AMU).

**Table 2:** Organisational characteristics participating hospitals.

	Total		University hospital		Teaching hospital		General hospital	
**Hospital type**	28		6	21.4%	14	50.0%	8	28.6%
**Number of beds**								
Internal medicine, mean (95% CI)*	65 (16–140)		92 (60–124)		69 (56–83)		36 (27–45)	
ED, Mean (95% CI)*	20 (7–36)		20 (15–25)		23 (18–27)		15 (11–19)	
**Present acute care facilities** ^**a**^								
No	4	14.3%	0	0.0%	1	7.1%	3	37.5%
Acute medical unit	19	67.9%	5	83.3%	9	64.3%	5	62.5%
Observation unit	5	17.9%	0	0.0%	4	28.6%	1	12.5%
Short stay unit*	3	3.6%	3	33.3%	0	0.0%	0	0.0%
**Maximum length of stay in acute care facility**								
No	1	3.6%	0	0.0%	1	7.1%	0	0.0%
24H	5	17.9%	1	16.7%	3	21.4%	1	12.5%
48H	17	64.3%	5	83.3%	8	64.3%	4	50.0%
72H	1	14.3%	0	0.0%	1	7.1%	0	0.0%
**Alternative acute care facilities outside the ED** ^**a**^								
Acute outpatient clinic^b^	23	82.1%	6	100%	12	85.7%	5	62.5%
Outpatient clinic treating physician^c^	16	57.1%	4	66.7%	9	64.3%	3	37.5%
Acute medical unit	9	32.1%	3	50,0%	4	28.6%	2	25.0%
Day care unit	6	21.4%	1	16.7%	2	14.3%	3	37.5%
Inpatient ward	7	25.0%	0	0.0%	5	35.7%	2	25.0%
Other^d^	4	14.3%	1	16.7%	2	14.3%	1	12.5%

*p ≤ 0.05.

^a^Multiple responses allowed, ^b^ Outpatient clinic for short-term assessment and management of urgent conditions.

^c^Possibility for urgent consultations with the patient’s treating specialist.

^d^Other: Acute service for oncology patients, sickle cell disease centre, specialist consult at the out-of-hours GP service.

Alternative acute care facilities were evaluated, with the acute outpatient clinic being the most commonly available, present in 23 hospitals (82.1%, Table 2). In a substantial proportion of these hospitals, patients could be seen by their treating physician. Other frequently mentioned alternative locations for acute care included inpatient wards and the outpatient day care unit.

### Patient characteristics

A total of 203 patients were included across 28 hospitals, with each hospital enrolling between three to fifteen patients (Table 3). Inclusion rates, reported by 13 hospitals, ranged from 44.4% to 100% of all internal medicine presentations with a median of 79.8%. Median age was 69 years (IQR 53–79), with university hospitals having the youngest cohort (60 years; IQR 43–76) and teaching hospitals the oldest (70 years; IQR 57–80). The majority of patients were aged 65–79 (33.0%), with no significant age group differences across hospital types. Overall, 51.2% of patients were female. The medication number of medication used was 5, and most patients had 1–2 comorbidities (47.8%). Functional status (CSF score) was similar across groups, with 11.8% of all patients categorised as severely frail (CFS 7–9). Additionally, the majority of patients were triaged U2 and U3, with significant differences between hospitals. In general hospitals patients were mostly triaged as U3 and in teaching hospitals mostly as U2. The most frequent presenting complaint was fever (31.0%), followed by general malaise (8.9%) (S1 Table).

**Table 3:** Patient characteristics.

	All patients	University hospital	Teaching hospital	General hospital
N=	203	40	124	39
**Age, median (IQR)**	69 (53–79)	60 (43–76)	70 (62–69)	68 (61–71)
**Age-groups**				
18-44	34 (16.7%)	10 (25.0%)	20 (16.1%)	4 (10.3%)
45-64	54 (26.6%)	11 (27.5%)	30 (24.2%)	13 (33.3%)
65-79	67 (33.0%)	12 (30.0%)	43 (34.7%)	12 (30.8%)
≥ 80	48 (23.6%)	7 (17.5%)	31 (25.0%)	10 (25.6%)
**Sex (F)**	104 (51.2%)	18 (45.0%)	66 (53.2%)	20 (51.3%)
**Medication** median number (IQR)	5.0 (2.0-10.0)	8.0 (4-13)	5.5 (2-9)	5.0 (1-10)
≥ 5, n= (%)	122 (60.1%)	26 (65.0%)	74 (59.7%)	22 (56.4%)
**Comorbidities**				
No*	32 (15.8%)	1 (2.5%)	25 (20.2%)	6 (15.4%)
1-2*	97 (47.8%)	15 (37.5%)	57 (46.0%)	25 (64.1%)
3-4*	48 (23.6%)	16 (40.0%)	27 (21.8%)	5 (12.8%)
≥ 5	26 (12.8%)	8 (20.0%)	15 (12.1%)	3 (7.7%)
**CFS**				
1–3	120 (59.1%)	23 (57.5%)	78 (62.9%)	19 (48.7%)
4–6	59 (29.1%)	12 (30.0%)	35 (28.2%)	12 (30.08%)
7-9	24 (11.8%)	5 (12.5%)	11 (8.9%)	8 (20.5%)
**Triage category**				
U0	3 (1.5%)	2 (5.0%)	1 (0.8%)	0 (0.0%)
U1	29 (14.3%)	8 (20.0%)	18 (14.5%)	3 (7.7%)
U2	105 (51.7%)	21 (52.5%)	70 (56.5%)	14 (35.9%)
U3*	59 (29.1%)	8 (20.0%)	29 (23.4%)	22 (56.4%)
U4-5	7 (3.4%)	1 (2.5%)	6 (4.8%)	0 (0.0%)
**LOS ED in minutes** median (IQR)	188 (142–240)	213 (160–286)	184 (141–241)	172 (132–209)

CFS: Clinical Frailty Scale, LOS: Length of Stay, ED: Emergency Department.

*p ≤ 0.05.

Half of the patients were referred by a GP (n = 106, 52.2%), with an internist consulted before referral in 62.6% (S2 Table). In university hospitals, referrals were more often made by in-hospital specialists (45.0%). Most referrals occurred during office hours (n = 144, 70.9%). Median LOS in the ED was significantly longer in university hospitals compared to general hospitals (213 minutes; IQR 160–286 vs. 172 minutes; IQR 132–209, p = 0.03). The majority of patients were admitted to the hospital (n = 123, 60.9%), and 73 patients (36.0%) were discharged home.

### Acute on chronic care

**Fig 1:**
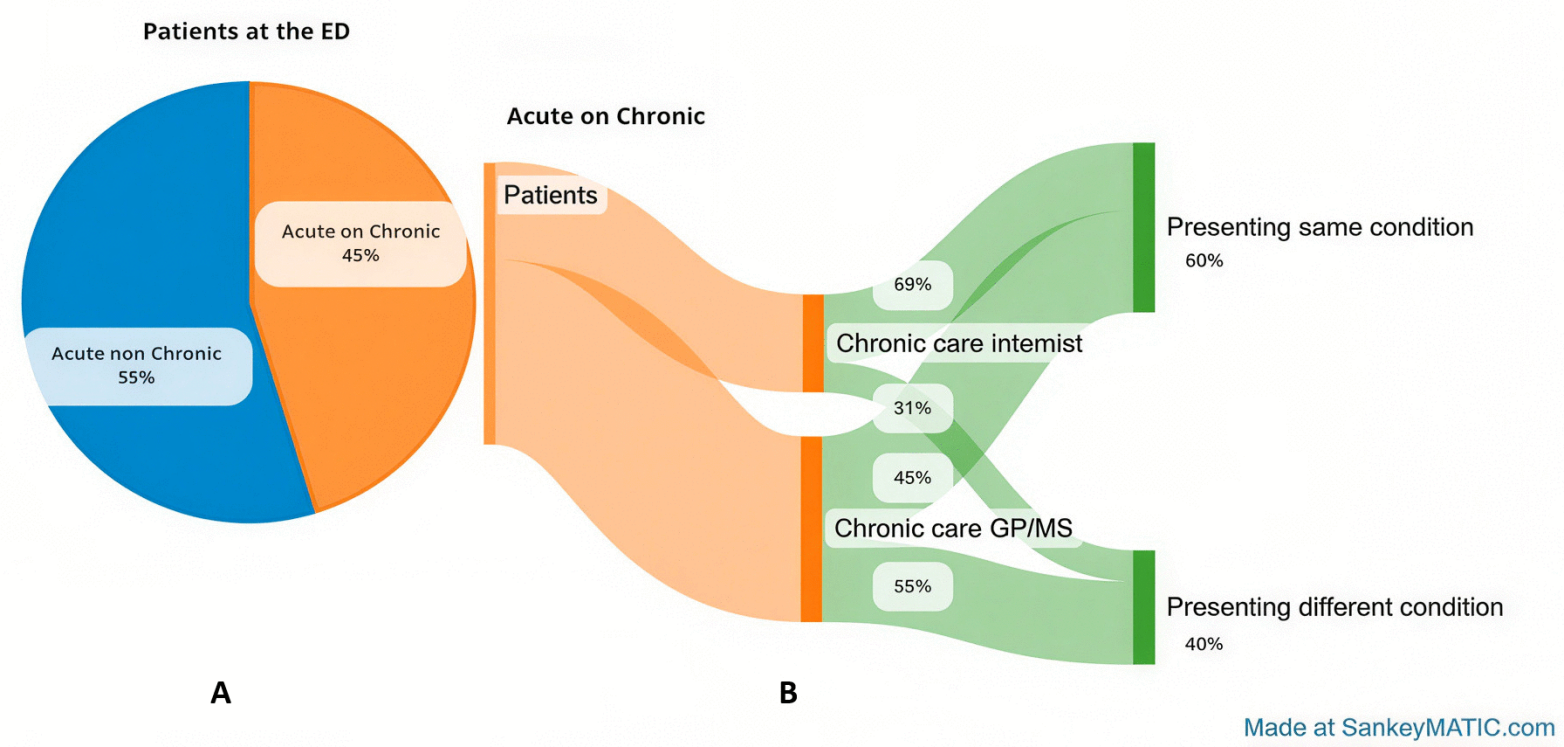
Distribution and care pathways of patients presenting to the emergency department (ED). Made at SankeyMATIC.com. GP: General Practitioner MS: Medical Specialist. **(A)** Pie chart showing the proportion of patients classified as *Acute non-chronic* (55%) versus *Acute on chronic* (45%). **(B)** Sankey diagram illustrating the flow of patients who are under chronic care of an internist or general practitioners/other medical specialists (GP/MS). Of these patients, 45% present with the same chronic condition, while 55% present with a different condition.

A large number of patients (n = 92; 45.3%) presented with a complication of a chronic condition (Fig 1). Of all patients already under follow-up by an internist (n = 80; 39.4%), 68.8% presented to the ED with the same chronic condition. Among patients receiving care for a chronic condition from a GP or other medical specialist (n = 151; 74.0%), 44.7% presented to the ED for internal medicine with the same condition they were being treated for by these physicians.

Most patients (n = 132, 62.0%) had no contact with hospital healthcare providers seven days before their ED-visit (S3 Table). Notably, patients at university hospitals were more likely to have prior hospital contact compared to other hospitals (51.2% vs. 34.1% and 37.5%, respectively). A small proportion of patients had either visited the ED (n = 9, 4.2%) or had been hospitalised (n = 11, 5.2%).

### Redirecting urgent care

Following clinical assessment, the primary responsible physician was asked to retrospectively determine whether the ED presentation could have been prevented within the current organisation of care, or whether the patient could theoretically have been redirected to an alternative acute care setting, irrespective of existing care structures (Table 4). In 49 cases (24.1%) the ED visit was considered preventable, and 81 patients (39.9%) were judged as eligible for redirection to alternative acute care settings. Among patients who presented with a complication of a chronic condition, 48.9% of ED visits were judged to have the potential to be redirected to an alternative setting (S4 Table).

**Table 4:** Possible prevention and redirection of ED presentation.

	Total	University hospital	Teaching hospital	General hospital
**Possible prevention of ED presentation?**				
No	154 (75.9%)	20 (64.5%)	97 (78.2%)	32(82.1%)
Yes	49 (24.1%)	11 (35.5%)	27 (21.8%)	7 (17.9%)
**Theoretical redirection to alternative setting possible?**				
No *	122 (60.1%)	18 (45.0%)	88 (71.0%)	16 (41.0%)
Yes *	81 (39.9%)	22 (55.0%)	36 (29.0%)	23 (59.0%)
**Alternative care settings** ^**a, b**^				
Acute outpatient clinic *	63 (77.8%)	13 (59.1%)	30 (83.3%)	20(87.0%)
Regular outpatient clinic	8 (9.9%)	2 (9.1%)	6 (16.7%)	0 (0.0%)
Acute Medical Unit	22 (27.2%)	8 (36.4%)	9 (25.0%)	5 (21.7%)
Inpatient ward*	12 (14.8%)	7 (31.8%)	2 (5.6%)	3 (13.0%)
Outpatient day care unit	3 (3.7%)	2 (9,1%)	1 (2.7%)	0 (0.0%)
GP	6 (7.4%)	2 (9.1%)	3 (8.3%)	1 (4.3%)
Other^c^	3 (3.7%)	0 (0.0%)	2 (5.6%)	1 (4.3%)
**Reasons for inability to redirect to alternative settings** ^**a,b**^				
Administration	0 (0.0%)	0 (0.0%)	0 (0.0%)	0 (0.0%)
No staffing capacity *	12 (14.8%)	1 (4.5%)	3 (8.3%)	8 (34.8%)
No available space *	12 (14.8%)	1 (4.5%)	2 (5.6%)	9 (39.1%)
Not structurally organised	47 (58.0%)	15 (68.2%)	17 (47.2%)	15 (65.2%)
Not a preference of the patient	3 (3.7%)	2 (9.1%)	1 (2.8%)	0 (0.0%)
Due to after-hours presentation	14 (17.3%)	3 (13.6%)	6 (16.7%)	5 (21.7%)
Referral via other healthcare provider	12 (14.8%)	2 (9.1%)	9 (25.0%)	1 (4.3%)
Other^d^	10 (12.3%)	1 (4.5%)	6 (16.7%)	3 (13.0%)

ED: Emergency Department, GP: General Practitioner.

*p ≤ 0.05.

^a^Multiple answers allowed ^b^ Proportion of patients considered eligible for redirection.

^c^Other, e.g., other clinic, procedure room, outpatient same day emergency care.

^d^Other, e.g., Inadequate inquiry of complaints by the GP, logistical reasons, no urgent lab tests at the acute outpatient clinic, and miscommunication among involved healthcare providers.

Most patients were considered suitable for redirection to an acute outpatient clinic (n = 63, 77.8%) or an AMU (n = 22, 27.2%). The main barrier to redirection was the lack of structurally organised acute care in alternative settings (n = 47, 58.0%), while only 14 patients (17.3%) were limited by out-of-hours presentation. Hospital contact within seven days prior to ED visit was more common among patients with preventable visits than non-preventable visits (n = 26; 53.1% vs. n = 45; 29.2%, p = 0.002), as was recent hospital admission (n = 6; 12.2% vs. n = 5; 3.2%, p = 0.02). Similarly, patients considered redirectable to alternative settings more often had prior hospital contact than non-redirectable patients (n = 39; 48.1% vs. n = 32; 26.2%, p = 0.001), and were more frequently referred by a hospital based specialist (n = 30; 37.0% vs n = 22; 18.0%, p = 0.001). An overview of this subgroup analysis is provided in S5 Table and S6 Table.

### Comparison of primary outcomes by hospital inclusion rate

A comparison between hospitals with an inclusion rate below 50% (n = 4) and those with a 100% (n = 3) inclusion rate showed no statistically significant differences in primary outcomes. The proportion of patients with a chronic disease was 46.8% versus 53.0% (p = 0.915), preventable visits 30.5% versus 24.0% (p = 0.54), and redirectable visits 50.8% versus 42.2% (p = 0.54).

## Discussion

In this nationwide observational flashmob study, we evaluated the characteristics of internal medicine ED presentations across 28 hospitals, focusing on chronic disease related visits and the potential for redirection to alternative acute care settings. We found that nearly half of the ED visits were due to an acute complication of a chronic condition, highlighting the overlap between acute and chronic care. In 24% of these cases, the visit was considered preventable and in nearly 40%, patients could potentially have been redirected to alternative acute care settings, as retrospectively judged by the primary responsible physician.

To the best of our knowledge, this is the first study investigating the hypothesis ‘acute care is chronic care’ in acute internal medicine patients. We found that almost half of the patients presented to the ED due to a complication of a chronic condition. In addition, 38.0% of patients had contact with a medical specialist seven days prior to their ED visit, including 4.2% who had visited the ED and 5.2% who had been admitted. Earlier research describes 7-day revisit rates between 2.6% and 14.4%, often driven by acute deterioration related to infection, cardiovascular disease or chronic kidney disease [13,14]. Together, these findings underscore the complexity of chronic care management across care settings and support the perspective that acute care increasingly serves as an extension of chronic care [4].

Furthermore, in addition to the high prevalence of chronic conditions, we found that patients presenting to the ED for internal medicine exhibited considerable care complexity, reflected in high rates of multimorbidity, polypharmacy, and frailty, further influenced by age and clinical urgency. These findings highlight the challenges of delivering acute care to patients with multiple and interacting health problems. This aligns with previous research indicating that acute medical patients often have complex health needs and multiple chronic conditions managed across specialties. For example, Buurman et al. reported that 87.8% of acutely hospitalised older adults had multimorbidity when both acute and chronic conditions were considered [15–17].

Our study found that 24% of ED visits were considered preventable based on retrospective assessment by healthcare professionals. In addition, approximately 40% of all ED visits, and nearly half of those related to a chronic condition, were considered theoretically manageable in a non-ED setting. These estimates should, however, be interpreted carefully, as the potential for redirection and preventability were assessed retrospectively after full ED evaluation and were based on the judgement of the primary responsible physician, which may vary between clinicians. Nevertheless, our findings are in line with previous studies in which ED physicians judged 31% and 23% of ED visits to be preventable [18,19]. As such, they provide a first indication that a substantial proportion of patients may be suitable for management in alternative acute care settings. This is particularly relevant because internal medicine patients constitute a substantial proportion of ED visits and their complex diagnostic work-up leads them to contribute disproportionately to ED crowding. Prevention or redirection of care in this group could therefore help alleviate system-wide congestion, even if they account for only one-quarter to one-fifth of the ED population. Interestingly, in both preventable and redirectable ED visits, patients were more likely to have had contact with the hospital prior to their ED presentation. These findings suggest that patients with preventable or redirectable ED visits are often already engaged in existing hospital-based care pathways. This is supported by a previous study showing an association between outpatient specialist contact up to one year prior to the ED visit and frequent ED use [20]. However, no studies have evaluated the relationship between hospital contact shortly before the ED visit and subsequent ED utilisation. Although based on relatively small numbers and derived from an exploratory 24-hour observational snapshot, our findings suggest that these prior contact moments may represent clinically relevant opportunities for earlier intervention. At the same time, they may also reflect failed attempts at redirection within existing care structures. Further research is warranted to understand the nature of recent hospital contacts and their potential role in preventing avoidable ED presentations and supporting alternative care pathways.

Lastly, 78% of patients suitable for redirection were judged potentially manageable in an acute outpatient clinic. This is particularly relevant given that most hospitals reported the availability of such a clinic. Several factors may contribute to this discrepancy. First, acute outpatient clinics may be insufficiently accessible or inadequately organised at the time of referral. This is further supported by the finding that redirectable patients were more often referred by hospital-based specialists, who are typically familiar with local acute care structures. Possible explanations include a lack of dedicated capacity for urgent cases, or logistical barriers such as limited timely access to diagnostics or insufficient staffing. Indeed, structural issues were the most frequently reported barrier to redirecting patients to alternative care settings. Second, it may be difficult to identify suitable patients for redirection in advance, before they present to the ED. This aligns with the findings from a review on the related concept of same day emergency care, which highlighted that both timely access to diagnostics and accurate identification of suitable patients were key factors for succes [21,22]. Moreover, longstanding reliance on the safety and rapid availability of extensive diagnostic facilities in the ED may further discourage redirection. While evidence on the safety and efficiency of acute outpatient clinics remains limited, the review showed promising results in safely delivering care outside the ED and reducing crowding [22]. To optimise the utilisation of alternative care settings, further research is needed to define the required diagnostic facilities and identify patient profiles most appropriate for such care.

Given these insights, a substantial proportion of ED visits may be preventable or redirectable. Earlier identification of such cases, through consistent criteria across clinicians and healthcare settings, together with proactive management and improved organisation and accessibility of alternative care settings, could help reduce unnecessary ED utilisation, particularly in times of increasing ED crowding and workforce constraints.

### Limitations

Several limitations should be considered when interpreting the findings of this study. First, the findings represent descriptive estimates from a single 24‑hour observational snapshot and may therefore not fully capture temporal variation. As an exploratory flashmob study, the results provide a cross-sectional overview of internal medicine ED presentations during the study period and should not be interpreted as generalisable measures of prevalence or healthcare system performance. Moreover although the study aimed to provide a representative overview of ED presentations, general hospitals were underrepresented in comparison to university and teaching hospitals. This imbalance may reflect the limited capacity of general hospitals to participate in time-intensive research such as this flashmob study, potentially introducing bias. However, we do not expect this to have a significant impact on the outcomes. Secondly, even though 28 hospitals participated, only 203 patients were included. Nevertheless, patients were recruited consecutively, and data on internal medicine presentations during the study period from 12 hospitals indicate that the majority of eligible patients were enrolled, with a median inclusion rate of 71.4% (range 44.4–100%). Moreover, data on presentations due to complications of chronic disease, preventable emergency visits, and redirectable visits did not differ between hospitals with inclusion rates below 50% and those with complete (100%) inclusion. Based on these data, selection bias is likely to be limited. The relatively modest number of patients per hospital likely reflects variations in recruitment hours, natural fluctuations in ED attendances, and the fact that not all patients provided consent. Furthermore, these assessments were made retrospectively by the primary responsible physician without predefined criteria, and interobserver variability was not assessed. Consequently, these estimates reflect subjective clinical judgement and may vary between clinicians and healthcare settings. In addition, this approach likely introduced hindsight, optimism, and post-diagnostic reassessment bias, as physicians had access to full diagnostic information at the time of judgement. This may have resulted in an overestimation of potential redirection, as it is often challenging to assess a patient’s condition and care needs prior to arrival at the ED. Nonetheless, this approach provides valuable insight into the potential scope for prevention and redirection of internal medicine ED visits and establishes a foundation for future studies to define criteria for safe prospective redirection as well as to explore opportunities for the development of alternative care pathways. Finally, the organisation of acute care within the Dutch healthcare systems may limit the generalisability of these findings to other healthcare settings with different referral structures or less gatekeeping and 24/7 access to GPs. However, given that demographic changes are occurring worldwide and ED crowding is a global challenge, our findings are still relevant internationally.

## Conclusion

This nationwide exploratory study suggests that acute internal medicine care in the ED is often an extension of chronic care, with many patients presenting due to complications of conditions for which they were already being treated. While alternative care pathways exist, their use remains limited, mainly due to organisational barriers. To ensure the right care in the right place at the right time, and safeguard the accessibility of EDs, future efforts must be focused on implementation and the efficient use of available resources.

## Supporting information

S1 Questionaire Organisational characteristics. (DOCX)

S2 Questionaire Patient characteristics. (DOCX)

S1 Table Presenting complaints in the ED. (DOCX)

S2 Table Referral to the ED and discharge location. (DOCX)

S3 Table Contact with hospital healthcare providers 7-days prior to ED visit. (DOCX)

S4 Table Possible prevention and redirection of ED presentation in acute on chronic patients. (DOCX)

S5. Table Sub-analysis: referral patterns and prior hospital contact in preventable ED visits. (DOCX)

S6. Table Sub-analysis: referral patterns and prior hospital contact in redirectable ED visits. (DOCX)
