# Supplementary material for: Chronic disease related emergency department presentations and potential for redirection to alternative acute care settings (“FOCUS” study): A nationwide flashmob study
Source: PLoS One. 2026 Jul 15;21(7):e0353157. doi: 10.1371/journal.pone.0353157 (PMC13372115; doi:10.1371/journal.pone.0353157)
Supplement: S1 Questionaire — (DOCX) [file pone.0353157.s001.docx]

**S1. Questionnaire 1: Organisational Characteristics (one time inventory)**

**Hospital Characteristics**

1. Hospital type
   1. Academic/University Hospital
   2. Top Clinical Hospital
   3. General Hospital
2. Number of Internal Medicine inpatient beds (including subspecialties: acute medicine, infectious diseases, geriatrics, vascular medicine, allergology, endocrinology, hematology, nephrology, and oncology) in the hospital (theoretical)? [Open question]
3. What are the opening hours of the Emergency Department (ED)? [Please check all that apply]
    a) 24/7
    b) During office hours (08:00-17:00)
    c) During the evening (17:00-23:00)
    d) Night ((23:00-08:00)
    e) Closed during the weekend
4. How many treatment bays are available in the Emergency Department? [Open question]
5. Is there a dedicated Acute Medical Unit?
   1. No
   2. Yes, an Acute Medical Unit
   3. Yes, an Observation Unit
   4. Yes, a Short Stay Unit
   5. Yes, other [open field]

If 5 is YES (i.e. b, c, d or e):
5.1.1 How many beds are available in this Acute Medical Unit (total theoretical capacity)? [Open field]

5.1.2 Is there a maximum length of stay for patients in this unit?
a) No
b) Yes, 6-12 hours
c) Yes, 12-24 hours
d) Yes, maximum 48 hours
e) Yes, maximum 72 hours

**Organisation of the ED**

1. Is there collaboration between the Emergency Department and the Out of Hours Primary Care (OOH-PC)?
   1. No
   2. Yes, collaboration exists and the OOH-PC is located in a different building than the hospital.
   3. Yes, OOH-PC and ED operate in parallel in the same building. The patient chooses.
   4. Yes, OOH-PC and ED operate in series. The patient is first assessed at the OOH-PC and referred to the ED if necessary.
   5. Yes, fully integrated OOH-PC and ED; joint triage takes place.
2. Is an Emergency Physician present* in the Emergency Department?
   *Presence: workplace on/near the ED, enabling participation in acute patient care at arrival.
   1. Not present
   2. Present 24/7
   3. Present during daytime and evenings (08:00-23:59)
   4. Other, namely [Open field]
3. Is an internist present* in the Emergency Department?
   *Presence: workplace on/near the ED, enabling participation in acute patient care at arrival.
   1. Not present
   2. Present 24/7
   3. Present during office hours (08:00-16:59)
   4. Present during daytime and evenings (08:00-23:59)
   5. Other, namely [Open field]

8.1 If present, is the internist an acute internist?

a) Yes
 b) No
 c) Other [Open field]

1. Are Internal Medicine residents present in the ED?
   *Presence: workplace on/near the ED, enabling participation in acute patient care at arrival.
   1. AIOS only
   2. ANIOS only
   3. Both AIOS and ANIOS
   4. No internal medicine residents

10. Who assesses patients referred to Internal Medicine in the ED? [Multiple answers possible]

1. ED physician
2. Internist
3. ED resident
4. Internal Medicine resident
5. General Practitioner resident (HAIO)

**Internal Medicine Hospital Care**

1. Who has decision-making authority regarding patients with an acute internal medicine care request? [Multiple answers possible]
2. Internist
3. ED physician
4. Internist and ED physician
5. Other [Open field]
6. Where can a patient with an acute internal medicine care request be assessed? [Multiple answers possible]
7. Acute outpatient clinic
8. Acute outpatient clinic treating physician
9. Emergency Department
10. Acute Medical Unit
11. Daycare unit
12. Inpatient ward
13. Other, namely [Open field]
