# Supplementary material for: Chronic disease related emergency department presentations and potential for redirection to alternative acute care settings (“FOCUS” study): A nationwide flashmob study
Source: PLoS One. 2026 Jul 15;21(7):e0353157. doi: 10.1371/journal.pone.0353157 (PMC13372115; doi:10.1371/journal.pone.0353157)
Supplement: S2 Questionaire — (DOCX) [file pone.0353157.s002.docx]

**S2. Questionnaire 2. Patient Information**

1. What is the patient’s age? [Open field]
2. What is the patient’s gender?
   1. Male
   2. Female
   3. Other
3. When did the patient present to the ED?
   1. During office hours (08:00-16:59)
   2. Evening (17:00-23:59)
   3. Night (00:00-07:59)
4. Who referred the patient to the hospital?
   1. General practitioner
   2. Hospital specialist
   3. External specialist
   4. Ambulance
   5. Self-referral
5. Which physician was consulted before referring the patient to the ED?
   1. Internal Medicine A(N)IOS
   2. ED A(N)IOS
   3. ED physician
   4. Internist
   5. Other [Open field]
6. What urgency category was assigned to the patient at triage?
   1. U0 – Red = acute
   2. U1 – Orange = very urgent
   3. U2 – Yellow = urgent
   4. U3 – Green = standard
   5. U3-U5 – Blue = non-urgent
7. Does the patient have known active comorbidities?
   1. No
   2. Yes, 1
   3. Yes, 2
   4. Yes, 3
   5. Yes, 4
   6. Yes, 5
   7. Yes, >5
8. How many medications* is the patient using at the time of the ED visit? Include: prescribed medications (daily/weekly), drops, inhaled meds, and regular OTC use such as paracetamol. Exclude: occasional OTC or complementary medicines. [Open answer, 0-20]
9. Clinical Frailty Scale (see Appendix 3)
   1. 1 Very fit
   2. 2 Fit
   3. 3 Managing well
   4. 4 Vulnerable
   5. 5 Mildly frail
   6. 6 Moderately frail
   7. 7 Severely frail
   8. 8 Very severely frail
   9. Terminally ill
10. Reason for ED visit:

- Acute bleeding
- Acute renal failure
- General deterioration
- Anemia
- Abdominal pain
- Electrolyte disorders
- COPD/asthma exacerbation
- Heart failure
- Intoxication
- Infection/sepsis
- Metabolic disorder
- Neutropenic fever
- Uncontrolled diabetes mellitus
- Thromboembolism
- Other

1. Is this an acute deterioration or complication of a chronic condition?
2. Yes
3. No
4. Is the patient under control/treatment by Internal Medicine?
5. Yes
6. No

12.1 *If yes:* Is the current ED visit related to the same condition for which the patient is receiving treatment from Internal Medicine?
a) Yes (related complaint) b) No (unrelated complaint)

13. Is the patient under active care by GP or another specialist?

1. Yes
2. No

13.1 *If yes:* Is the current ED visit related to the same condition for which the patient is receiving treatment from the GP or another specialist?
 a) Yes
 b) No

14. Has the patient had contact with hospital care providers in the past 7 days?

1. Yes, phone contact
2. Yes, outpatient visit
3. Yes, ED visit
4. Yes, inpatient admission
5. No contact

15. Could this ED visit have been prevented?

1. Yes
2. No

16. Could the patient theoretically have been seen elsewhere?

1. No
2. Yes, emergency outpatient clinic
3. Yes, regular outpatient clinic
4. Yes, acute admission ward
5. Yes, inpatient ward
6. Yes, other [Open field]

16.1 If yes, why was the patient not seen elsewhere
 a) No staff capacity
 b) Not structurally organized
 c) No space
 d) Time of day (evening/night)
 e) Too busy/no time
 f) Patient preference
 g) Referred via another provider
 h) Not assessed at triage
 i) Administrative issues (registration/insurance)
 j) Other [Open field]

17. Length of stay in ED (minutes)? [Open answer]

18. Discharge destination after ED visit:

1. Home with outpatient follow-up
2. Home without follow-up
3. Admission to acute ward
4. Admission to hospital ward
5. ICU/MC admission
6. Nursing home
7. Rehabilitation
8. Deceased in ED
9. Other [Open field]
