# Supplementary material for: Chronic disease related emergency department presentations and potential for redirection to alternative acute care settings (“FOCUS” study): A nationwide flashmob study
Source: PLoS One. 2026 Jul 15;21(7):e0353157. doi: 10.1371/journal.pone.0353157 (PMC13372115; doi:10.1371/journal.pone.0353157)
Supplement: S1 Table — (DOCX) [file pone.0353157.s003.docx]

**Supplementary tables S1**

**S1 Table: Presenting complaints in the ED**

|  | **Total  n = 203** | **University**  **Hospital n = 40** | **Teaching** **hospital n = 124** | **General** **hospital n = 39** |
| --- | --- | --- | --- | --- |
| Acute bleeding | 2 (1.0%) | 1 (2.5%) | 0 (0%) | 1 (2.6%) |
| Acute kidney failure | 3 (1.5%) | 1 (2.5%) | 1 (0.8%) | 1 (2.6%) |
| General decline | 18 (8.9%) | 4 (10.0%) | 11 (8.9%) | 3 (7.7%) |
| Anemia | 10 (4.9%) | 3 (7.5%) | 5 (4.0%) | 2 (5.1%) |
| Abdominal complaints | 19 (9.3%) | 4(10.0%) | 11 (8.9%) | 4 (10.2%) |
| Electrolyte disturbances | 9 (4.4%) | 0 (0.0%) | 7 (5.6%) | 2 (5.1%) |
| Heart failure | 2 (1.0%) | 0 (0.0%) | 2 (1.6%) | 0 (0.0%) |
| Intoxication | 2 (1.0%) | 0 (0.0%) | 2 (1.6%) | 0 (0.0%) |
| Infection/Sepsis | 63 (31.0%) | 15 (37.5%) | 34 (27.4%) | 14 (35.9%) |
| Metabolic disorder | 4 (2.0%) | 0 (0.0%) | 3 (2.4%) | 1 (2.6%) |
| Neutropenic fever | 4 (2.0%) | 1 (2.5%) | 2 (1.6%) | 1 (2.6%) |
| Uncontrolled diabetes | 4 (2.0%) | 2 (5.0%) | 2 (1.6%) | 0 (0.0%) |
| Pain in malignancy | 4 (2.0%) | 0 (0.0%) | 4 (3.2%) | 0 (0.0%) |
| Tromboembolism/  Thrombosis | 12 (5.9%) | 1 (2.5%) | 7 (5.6%) | 4 (10.3%) |
| Hypertension | 7 (3.4%) | 0 (0.0%) | 6 (4.8%) | 1 (2.6%) |
| Syncope | 5 (2.5%) | 0 (0.0%) | 5 (4.0%) | 0 (0.0%) |
| Neurological complaints | 5 (2.5%) | 1 (2.5%) | 4 (3.2%) | 0 (0.0%) |
| Chest pain/dyspnea | 6 (3.0%) | 1 (2.5%) | 5 (4.0%) | 0 (0.0%) |
| Allergic reaction | 6 (3.0%) | 1 (2.5%) | 4 (3.2%) | 1 (2.6%) |
| Other^a^ | 18 (8.9%) | 5 (12.5%) | 9 (7.3%) | 4 (10.3%) |

^a^ Other: luxation probe, sickle cell crisis, pain in neck/axilla, palpable swelling in the neck, suspected temporal arteritis, urinary retention, jaundice, suspected leukemia, urinary retention)
