# Supplementary material for: Chronic disease related emergency department presentations and potential for redirection to alternative acute care settings (“FOCUS” study): A nationwide flashmob study
Source: PLoS One. 2026 Jul 15;21(7):e0353157. doi: 10.1371/journal.pone.0353157 (PMC13372115; doi:10.1371/journal.pone.0353157)
Supplement: S2 Table — (DOCX) [file pone.0353157.s004.docx]

**S2 Table Referral to the ED and discharge location**

|  | **Total n = 203** | **University**  **Hospital n = 40** | **Teaching**  **Hospital**  **n = 124** | **General**  **Hospital**  **n = 39** | **p-value** |
| --- | --- | --- | --- | --- | --- |
| **Referring physician** |  |  |  |  |  |
| General Practitioner (GP) | 106 (52.2%) | 15 (37.5%) | 65 (52.4%) | 26 (66.7%) | 0.034 |
| Ambulance | 30 (14.8%) | 4 (10.0%) | 24 (19.4%) | 2 (5.1%) | 0.059 |
| Hospital specialist within the hospital | 52 (25.6%) | 18 (45.0%) | 26 (21.0%) | 8 (20.5%) | 0.007 |
| A specialist outside the hospital | 8 (3.9%) | 0 (0%) | 5 (4.0%) | 3 (7.7%) | 0.213 |
| Self-referral | 7 (3.4%) | 3 (7.5%) | 4 (3.2%) | 0 (0.0%) | 0.184 |
| **Consulted physician to refer to the ED** | | | | | |
| Internist | 127 (62.6%) | 20 (50.0%) | 80 (64.5%) | 27 (69.2%) | 0.162 |
| Emergency Physician | 27 (13.3%) | 3 (7.5%) | 19 (15.3%) | 5 (12.8%) | 0.446 |
| Resident internal medicine | 22 (10.8%) | 12 (30.0%) | 9 (7.3%) | 1 (2.6%) | <0.001 |
| Resident ED | 2 (1.0%) | 0 (0.0%) | 1 (0.8%) | 1 (2.6%) | 0.488 |
| Other^a^ | 25 (12.3%) | 5 (12.5%) | 15 (12.1%) | 5 (12.8%) | 0.992 |
| **Time of presentation** |  |  |  |  | 0.868 |
| During office hours (08:00-16:59) | 144 (70.9%) | 31 (77.5%) | 86 (69.4%) | 27 (69.2%) |  |
| In the evening (17:00-23:59) | 52 (25.6%) | 8 (20.0%) | 33 (26.6%) | 11 (28.2%) |  |
| At night (00:00-07:59) | 7 (3.4%) | 1 (2.5%) | 5 (4.0%) | 1 (2.6%) |  |
| **Discharge location** |  |  |  |  |  |
| Home with follow-up | 29 (14.3%) | 9 (22.5%) | 15 (12.1%) | 5 (13.2%) | 0.26 |
| Home without follow/up | 44 (21.7%) | 7 (17.5%) | 26 (21.0%) | 11 (28.9%) | 0.44 |
| Hospital admission | 123 (60,9%) | 24 (60.0%) | 80 (64.5%) | 19 (48.7%) | 0.19 |
| Admission to ICU/ICU | 1 (0.5%) | 0 (0%) | 0 (0%) | 1 (2.6%) | 0.11 |
| Nursing home | 3 (1.5%) | 0 (0%) | 3 (2.4%) | 0 (0%) | 0.38 |
| Other^b^ | 2 (1.0%) | 0 (0%) | 0(0%) | 2 (5.3%) | 0.01 |

*^a^Other: Physician assistant, specialised nurse, self-referral*
*^b^Other: Primary care bed or observation unit*
