# Supplementary material for: Chronic disease related emergency department presentations and potential for redirection to alternative acute care settings (“FOCUS” study): A nationwide flashmob study
Source: PLoS One. 2026 Jul 15;21(7):e0353157. doi: 10.1371/journal.pone.0353157 (PMC13372115; doi:10.1371/journal.pone.0353157)
Supplement: S3 Table — (DOCX) [file pone.0353157.s005.docx]

**S3 Table Contact with hospital healthcare providers 7-days prior to ED visit**

|  | **Total** | **University** **hospital** | **Teaching** **hospital** | **General** **hospital** | **p-value** |
| --- | --- | --- | --- | --- | --- |
| No | 132 (62.0%) | 20 (48.8%) | 87 (65.9%) | 25 (62.5%) | 0.142 |
| Yes, called the ED | 27 (12.7%) | 10 (24.4%) | 14 (10.6%) | 3 (7.5%) | 0.038 |
| Yes, outpatient clinic contact | 24 (11.3%) | 5 (12.2%) | 11 (8.3%) | 8 (20.0%) | 0.121 |
| Yes, ED visit | 9 (4.2%) | 2 (4.9%) | 5 (3.8%) | 2 (5.0%) | 0.921 |
| Yes, hospital admission | 11 (5.2%) | 3 (7.3%) | 7 (5.3%) | 1 (2.5%) | 0.615 |
