# Supplementary material for: Chronic disease related emergency department presentations and potential for redirection to alternative acute care settings (“FOCUS” study): A nationwide flashmob study
Source: PLoS One. 2026 Jul 15;21(7):e0353157. doi: 10.1371/journal.pone.0353157 (PMC13372115; doi:10.1371/journal.pone.0353157)
Supplement: S4 Table — (DOCX) [file pone.0353157.s006.docx]

**S4 Possible prevention and redirection of ED presentation in acute on chronic patients**

|  | **Total (n=92)** | **University Hospital (n = 21)** | **Teaching Hospital (n = 57)** | **General Hospital (n = 14)** |
| --- | --- | --- | --- | --- |
| **Possible prevention of ED presentation?** | | | | |
| No | 65 (70.7%) | 12 (57.1%) | 43 (75.4%) | 10 (71.4%) |
| Yes | 27 (29.3%) | 9 (42.9%) | 14 (24.6%) | 4 (28.6%) |
| **Theoretical redirection to alternative setting possible?** | | | | |
| No | 47 (51.1%) | 7 (33.3%) | 34 (59.6%) | 6 (42.9%) |
| Yes | 45 (48.9%) | 14 (66.7%) | 23 (40.4%) | 8 (57.1%) |
