# Supplementary material for: Chronic disease related emergency department presentations and potential for redirection to alternative acute care settings (“FOCUS” study): A nationwide flashmob study
Source: PLoS One. 2026 Jul 15;21(7):e0353157. doi: 10.1371/journal.pone.0353157 (PMC13372115; doi:10.1371/journal.pone.0353157)
Supplement: S5. Table — (DOCX) [file pone.0353157.s007.docx]

**S5 Table Sub-analysis: referral patterns and prior hospital contact in preventable ED visits**

|  | **Preventable** | **Non-preventable** | **p-value** | **Acute on chronic^a^** | **Non-Acute on chronic^a^** | **p-value** |
| --- | --- | --- | --- | --- | --- | --- |
| **Referring physician** |  |  |  |  |  |  |
| General Practitioner (GP) | 24 (49.0%) | 82 (53.2%) | 0.602 | 10 (37.0%) | 14 (63.3%) | 0.064 |
| Ambulance | 4 (8.2%) | 26 (16.9%) | 0.134 | 2 (7.4%) | 2 (9.1%) | 0.83 |
| A specialist within the hospital | 16 (32.7%) | 36 (23.4%) | 0.195 | 12(44.4%) | 4 (18.2%) | 0.051 |
| A specialist outside the hospital | 1 (2.0%) | 7 (4.5%) | 0.433 | 1 (3.7%) | 0 (0.0%) | 0.362 |
| Self-referral | 4 (8.2%) | 3 (1.9%) | 0.038 | 2 (7.4%) | 2 (9.1%) | 0.83 |
| **Hospital contact <7 days** |  |  |  |  |  |  |
| No | 23 (46.9%) | 109 (70.8%) | 0.002 | 9 (33.3%) | 14 (63.3%) | 0.035 |
| Yes, by telephone | 10 (20.4%) | 17 (11.0%) | 0.093 | 9 (33.3%) | 1 (4.5%) | 0.013 |
| Yes, outpatient clinic | 9 (18.4%) | 15 (9.7%) | 0.103 | 5 (18.5%) | 4 (18.2%) | 0.976 |
| Yes, ED visit | 1 (2.0%) | 8 (5.2%) | 0.350 | 0 (0.0%) | 1 (4.5%) | 0.263 |
| Yes, hospital admission | 6 (12.2%) | 5 (3.2%) | 0.015 | 4 (14.8%) | 2 (9.1%) | 0.543 |

^a^ Only includes patients with preventable ED visits, stratified by acute-on-chronic presentation
