# Supplementary material for: Chronic disease related emergency department presentations and potential for redirection to alternative acute care settings (“FOCUS” study): A nationwide flashmob study
Source: PLoS One. 2026 Jul 15;21(7):e0353157. doi: 10.1371/journal.pone.0353157 (PMC13372115; doi:10.1371/journal.pone.0353157)
Supplement: S6. Table — (DOCX) [file pone.0353157.s008.docx]

**S6 Table Sub-analysis: referral patterns and prior hospital contact in redirectable ED visits.**

|  | **Redirectable** | **Non-redirectable** | **p-value** | **Acute on chronic^a^** | **Non-Acute on chronic^a^** | **p-value** |
| --- | --- | --- | --- | --- | --- | --- |
| **Referring physician** |  |  |  |  |  |  |
| General Practitioner (GP) | 40 (49.4%) | 66 (54.1%) | 0.51 | 17 (37.8%) | 23 (63.9%) | 0.02 |
| Ambulance | 5 (6.2%) | 25 (20.5%) | 0.005 | 1 (2.2%) | 4 (11.1%) | 0.099 |
| A specialist within the hospital | 30 (37.0%) | 22 (18.0%) | 0.002 | 22 (48.9%) | 8 (22.2%) | 0.014 |
| A specialist outside the hospital | 2 (2.5%) | 6 (4.9%) | 0.38 | 2 (4.4%) | 0 (0%) | 0.2 |
| Self-referral | 4 (4.9%) | 3 (2.5%) | 0.343 | 3 (6.7%) | 1 (2.8%) | 0.13 |
| **Hospital contact <7 days** |  |  |  |  |  |  |
| No | 42 (51.9%) | 90 (73.8%) | 0.001 | 16 (35.6%) | 26 (72.2%) | 0.001 |
| Yes, by telephone | 16 (19.8%) | 11 (9.0%) | 0.027 | 15 (33.3%) | 1 (2.8%) | <0.001 |
| Yes, outpatient clinic | 16 (19.8%) | 8 (6.6%) | 0.004 | 9 (20.0%) | 7 (19.4%) | 0.950 |
| Yes, ED visit | 2 (2.5%) | 7 (5.7%) | 0.268 | 1 (2.2%) | 1 (2.8%) | 0.873 |
| Yes, hospital admission | 5 (6.2%) | 6 (4.9%) | 0.699 | 4 (8.9%) | 1 (2.8%) | 0.256 |
|  |  |  |  |  |  |  |

^a^ Only includes patients with redirectable ED visits, stratified by acute-on-chronic presentation
