# Supplementary material for: Chronic disease related emergency department presentations and potential for redirection to alternative acute care settings (“FOCUS” study): A nationwide flashmob study
Source: PLoS One. 2026 Jul 15;21(7):e0353157. doi: 10.1371/journal.pone.0353157 (PMC13372115; doi:10.1371/journal.pone.0353157)
Supplement: Attachment — Submitted filename: FOCUS_Response_to_Reviewers_auresp_2.docx [file pone.0353157.s012.docx]

**Response to reviewers**

Dear Editor and Reviewers,

We would like to thank you for your careful evaluation of the revised manuscript and for your additional constructive comments. We appreciate the opportunity to further improve our work.

We have revised the manuscript accordingly and have addressed all remaining comments. A detailed point-by-point response is provided below.

We hope that the revised manuscript satisfactorily addresses the reviewers’ concerns and is now suitable for publication.

Thank you again for your consideration.

Sincerely,

M.N. Körnmann & E.M. Mols
On behalf of all authors

[m.n.koernmann@amsterdamumc.nl](mailto:m.n.koernmann@amsterdamumc.nl)

# **Comments** **1.1 – Interpretation of Flashmob Design**

*The authors have appropriately acknowledged that the study represents a 24-hour observational snapshot. However, the implications of this design should be more explicitly reflected in the interpretation of the findings.*

*The results should be consistently framed as* ***exploratory and descriptive estimates****, rather than as generalisable measures of prevalence or system performance.*

**Response:**
We thank the reviewer for this comment. We agree that the implications of the flashmob design should be reflected consistently throughout the interpretation of the findings. In response, we have further revised the Discussion, Limitations, and Conclusion sections to more explicitly frame the results as exploratory and descriptive estimates derived from a 24-hour observational snapshot.

**Changes in manuscript:**

- Discussion:
  “Although based on relatively small numbers and derived from an exploratory 24-hour observational snapshot, our findings suggest that these prior contact moments may represent clinically relevant opportunities for earlier intervention.” (Line 306)
- Limitations: “As an exploratory flashmob study, the results provide a cross-sectional overview of internal medicine ED presentations during the study period and should be interpreted descriptive estimates of prevalence or healthcare system performance.”
  (Lines 340-343)
- Conclusion:
  “This nationwide exploratory study suggests that acute internal medicine care in the ED is often an extension of chronic care, with many patients presenting due to complications of conditions for which they were already being treated. While alternative care pathways exist, their use remains limited, mainly due to organisational barriers.”  (Line 370)

### **1.2 Subjectivity and Definition of Preventability and Redirection Outcomes**

*The manuscript now acknowledges that preventability and redirection were assessed retrospectively by the treating physician.*

*However, a more fundamental methodological concern remains. These key outcomes appear to be based solely on the* ***subjective judgement of the primary responsible physician****, without predefined criteria, standardised definitions, or assessment of interobserver reliability. As currently presented, it is unclear how consistently these outcomes could be applied across different clinicians and settings.*

**Response:**
We thank the reviewer for this important comment and agree that the subjective nature of these assessments represents an important methodological limitation. Because preventability and redirection potential were based on the retrospective judgement of the primary responsible physician without predefined criteria or assessment of interobserver variability, the resulting estimates may vary between clinicians and may not be directly generalisable across healthcare settings. We have therefore strengthened the Discussion and Limitations sections to more explicitly acknowledge these limitations and to emphasise that future research is needed to develop and evaluate standardised criteria for prevention and redirection.

**Changes in manuscript:**

- Discussion
  “These estimates should, however, be interpreted carefully, as the potential for redirection and preventability were  assessed retrospectively after full ED evaluation and were based on the judgement of the primary responsible physician, which may vary between clinicians. Nevertheless,  our findings are in line with previous studies in which ED physicians judged 31% and 23% of ED visits to be preventable [18, 19]. As such, they provide a first indication that a substantial proportion of patients may be suitable for management in alternative acute care settings.”(Lines 290– 296)
- Discussion
  “Given these insights, a substantial proportion of ED visits may be preventable or redirectable. Earlier identification of such cases, through consistent criteria across clinicians and healthcare settings, together with proactive management and improved organisation and accessibility of alternative care settings, could help reduce unnecessary ED utilisation, particularly in times of increasing ED crowding and workforce constraints.” (Lines 331 – 335)
- Limitations:
  Furthermore, these assessments were made retrospectively by the primary responsible physician without predefined criteria, and interobserver variability was not assessed. Consequently, these estimates reflect subjective clinical judgement and may vary between clinicians and healthcare settings. In addition, this approach likely introduced hindsight, optimism, and post‑diagnostic reassessment bias, as physicians had access to full diagnostic information at the time of judgement. This may have resulted in an overestimation of potential redirection, as it is often challenging to assess a patient's condition and care needs prior to arrival at the ED. Nonetheless, this approach provides valuable insight into the potential scope for prevention and redirection of internal medicine ED visits and establishes a foundation for future studies to define criteria for safe prospective redirection as well as to explore opportunities for the development of alternative care pathways. (Lines 355-368)
