# Supplementary material for: Chronic disease related emergency department presentations and potential for redirection to alternative acute care settings (“FOCUS” study): A nationwide flashmob study
Source: PLoS One. 2026 Jul 15;21(7):e0353157. doi: 10.1371/journal.pone.0353157 (PMC13372115; doi:10.1371/journal.pone.0353157)
Supplement: Attachment — Submitted filename: FOCUS Response to Reviewers.docx [file pone.0353157.s011.docx]

**Response to reviewers**

Dear editor and reviewers,

We would like to sincerely thank you and the reviewers for your careful evaluation of our manuscript and for the constructive and insightful comments provided. We greatly appreciate the time and effort invested in reviewing our work. The feedback has helped us to significantly improve the clarity, transparency, and overall quality of the manuscript.

We have revised the manuscript thoroughly in accordance with all editorial and reviewer comments. Below, we provide a structured summary of the major revisions made.

We believe that these revisions have substantially strengthened the manuscript and addressed all concerns raised. We hope that the revised version meets the journals standards for publication.

Thank you again for your consideration.

Sincerely,

M.N. Körnmann & E.M. Mols

On behalf of all authors

m.n.koernmann@amsterdamumc.nl

# **EDITOR COMMENTS** **E1 – Methods clarity (study design, physician question, variables)**

*The reporting of the methods section needs some improvement. Please clarify in the study design that the study includes two steps, the first assessing patients and the second questioning doctors. Please provide the exact question asked to doctors. Please provide a clear paragraph of major variables collected with definitions especially for the variables included in the tables (what is U0, U1,…etc?).*

**Response:**

We thank the reviewer for this constructive comment. We agree that further clarification of the Methods section improves transparency and reproducibility.

In response, we have revised the study design section to more clearly describe the two-step approach of the study: (1) prospective inclusion and assessment of patients presenting to the ED, and (2) subsequent data collection from treating physicians regarding preventability and potential redirection of care.

We have also added the exact question(s) posed to physicians in the manuscript to improve clarity regarding how these outcomes were assessed and included the full questionnaires as Supplementary Material to allow readers to review all items in detail.

Furthermore, we have expanded the description of the collected variables and have provided clearer definitions of key variables used in the tables. In particular, we have clarified the meaning of urgency categories (e.g. U0–U5) and other core variables. To enhance readability, an overview of the main variables and their definitions has been added in tabular form in the Methods section.

These revisions aim to improve the clarity and interpretability of the study methodology.

**Changes in manuscript:**

- Methods → Data collection (Lines 142–156)
- Methods → Definitions and variables of interest (Lines 158–160)

### **E2 – PLOS formatting**

*Please ensure that your manuscript meets PLOS ONE's style requirements, including those for file naming. The PLOS ONE style templates can be found at.*

**Response:**

We thank the reviewer for this constructive comment. The manuscript has been fully reformatted according to PLOS ONE guidelines.

**Changes in manuscript:**

- Entire manuscript

### **E3 – Ethics statement**

*Please include your full ethics statement in the ‘Methods’ section of your manuscript file. In your statement, please include the full name of the IRB or ethics committee who approved or waived your study, as well as whether or not you obtained informed written or verbal consent. If consent was waived for your study, please include this information in your statement as well.*

**Response:**

A complete ethics statement has been added, including committee name and consent procedures.

**Changes in manuscript:**

- Methods → Ethics (Lines 103–105)

### **E4 – Consortium authorship**

*One of the noted authors is a group or consortium [on behalf of the ORCA (Onderzoeks Consortium Acute Geneeskunde) Acute Medicine Research Consortium and local investigators].*

*In addition to naming the author group, please list the individual authors and affiliations within this group in the acknowledgments section of your manuscript. Please also indicate clearly a lead author for this group along with a contact email address*

**Response:**
We have added all consortium members and affiliations in the Acknowledgments section and identified the lead author with contact details.

**Changes in manuscript:**

- Acknowledgments (Lines 386–403)

### **E5 – Data availability (restrictions explanation)**

*We note that you have indicated that there are restrictions to data sharing for this study. For studies involving human research participant data or other sensitive data, we encourage authors to share de-identified or anonymized data. However, when data cannot be publicly shared for ethical reasons, we allow authors to make their data sets available upon request. For information on unacceptable data access restrictions, please see* [*http://journals.plos.org/plosone/s/data-availability#loc-unacceptable-data-access-restrictions*](http://journals.plos.org/plosone/s/data-availability#loc-unacceptable-data-access-restrictions)*.*

**Response:**

We thank the editor for raising this point. We understand the importance of open data availability for transparency, reproducibility, and scientific progress. Unfortunately, explicit consent for public data sharing was not obtained in the study protocol or informed consent materials. Public release of the dataset is therefore not permitted under the arrangements established with our institutional Medical Ethics Review Committee of the Máxima Medical Center and the local Privacy Officer. This is especially relevant given the 24hour snapshot design and the relatively small number of patients in total and also per hospital.

However, anonymised data may be made available upon reasonable request. Requests can be directed to the corresponding author or to the local Medical Ethics Review Committee. Each request will be evaluated individually, considering necessity, proportionality, data minimisation, and the implementation of appropriate safeguards. Data is available via the corresponding author Michelle Körnmann ([m.n.koernmann@amsterdamumc.nl](mailto:m.n.koernmann@amsterdamumc.nl)) or the Medical Ethics Review Committee
of Maxima Medical Center ([metc@mmc.nl](mailto:metc@mmc.nl)) upon reasonable request.

**Changes in manuscript:**

- Data Availability Statement
  “Data is available via the corresponding author Michelle Körnmann ([m.n.koernmann@amsterdamumc.nl](mailto:m.n.koernmann@amsterdamumc.nl)) or the Medical Ethics Review Committee ([metc@mmc.nl](mailto:metc@mmc.nl)) upon reasonable request. All patients provided informed consent for the collection and use of their data for the purposes of this study. However, consent for public data sharing was not obtained. This approach was reviewed and approved by the local medical ethics committee and privacy officer in accordance with applicable ethical guidelines and regulations.”

### **E6 – Data sharing policy compliance**

*All PLOS journals now require all data underlying the findings described in their manuscript to be freely available to other researchers, either 1. In a public repository, 2. Within the manuscript itself, or 3. Uploaded as supplementary information.*
*This policy applies to all data except where public deposition would breach compliance with the protocol approved by your research ethics board. If your data cannot be made publicly available for ethical or legal reasons (e.g., public availability would compromise patient privacy), please explain your reasons on resubmission and your exemption request will be escalated for approval.*

**Response:**
As mentioned above, explicit consent for public data sharing was not obtained in the study protocol or informed consent materials. Public release of the dataset is therefore not permitted under the arrangements established with our institutional Medical Ethics Review Committee of the Máxima Medical Center and the local Privacy Officer.

**Changes in manuscript:**

- Data Availability Statement:
  “Data is available via the corresponding author Michelle Körnmann ([m.n.koernmann@amsterdamumc.nl](mailto:m.n.koernmann@amsterdamumc.nl)) or the Medical Ethics Review Committee ([metc@mmc.nl](mailto:metc@mmc.nl)) upon reasonable request. All patients provided informed consent for the collection and use of their data for the purposes of this study. However, consent for public data sharing was not obtained. This approach was reviewed and approved by the local medical ethics committee and privacy officer in accordance with applicable ethical guidelines and regulations.”

### **E7 – Funding role**

*Please state what role the funders took in the study. If the funders had no role, please state: "The funders had no role in study design, data collection and analysis, decision to publish, or preparation of the manuscript. "If this statement is not correct you must amend it as needed. Please include this amended Role of Funder statement in your cover letter; we will change the online submission form on your behalf.*

**Response:**

We confirm that the funder had no role and added the required statement.

**Changes in manuscript:**

- Funding section (Lines 376–377)

### **E8 – Title consistency**

*Please amend either the title on the online submission form (via Edit Submission) or the title in the manuscript so that they are identical.*

**Response:**

The title has been harmonized across all submission components.

**Changes in manuscript:**

- Title page

### **E9 – Authorship additions**

*Please amend your authorship list in your manuscript file to include author Michelle Noël Körnmann, Elisabeth Margaretha Mols, Harm R. Haak, Jelmer Alsma, Patricia Stassen, Karin Kaasjager, Geert Groeneveld, Marjolein N.T. Kremers.*

**Response:**

The requested authors have been added.

**Changes in manuscript:**

- Title page / author list

### **E10 – Submission metadata authors**

*Please amend the manuscript submission data (via Edit Submission) to include author E.M. Mols, M.N. Körnmann, H.R. Haak, J. Alsma, P. Stassen, H.A.H. Kaasjager, G.H. Groeneveld, M.N.T. Kremers.*

**Response:**

The submission system has been updated accordingly.

**Changes:**

- Submission system (no manuscript change)

### **E11 – Figure 1 copyright**

*We note that Figure 1 in your submission contain copyrighted images. All PLOS content is published under the Creative Commons Attribution License (CC BY 4.0), which means that the manuscript, images, and Supporting Information files will be freely available online, and any third party is permitted to access, download, copy, distribute, and use these materials in any way, even commercially, with proper attribution.*

**Response:**
We thank the Editor for this comment. We would like to clarify that Figure 1 is an original figure created by the authors, using the web-based tool SankeyMATIC. The figure is based entirely on our own study data and does not contain or reproduce any previously published or copyrighted material.

SankeyMATIC is a free tool that generates diagrams locally in the user’s browser, and its terms of use explicitly allow unrestricted use of generated figures without attribution or licensing restrictions. Therefore, no third-party copyright applies to Figure 1.

To improve transparency, we have added a statement in the figure caption indicating that the diagram was created by the authors using SankeyMATIC. In addition, we would like to include our syntax used for developing the figure on this site here:

// Enter Flows between Nodes, like this:

//	Source [AMOUNT] Target

Patients [123] No internist care

Patients [80] Chronic care internis

Chronic care internist [55] Presenting same condition

Chronic care internist [25] Presenting different condition

Patients [52] No care GP/MS

Patients [151] Chronic care GP/MS

Chronic care GP/MS [83] Presenting same condition

Chronic care GP/MS [68] Presenting different condition

**Changes in manuscript:**

- Figure 1 (Lines 218): added “made at SankeyMATIC.com".

### **E12 – Table 3 reference**

*We note you have included a table to which you do not refer in the text of your manuscript. Please ensure that you refer to Table 3 in your text; if accepted, production will need this reference to link the reader to the Table.*

**Response:**

This table is now explicitly referenced in the text.

### **E13 – Table 5 inclusion**

*Please include a copy of Table 5 which you refer to in your text on page 12.*

**Response:**

The reference to “Table 5” was a typographical error. No additional table was intended, and the incorrect reference has been removed from the manuscript.

### **E14 – Supporting information anonymisation**

*We note that there is identifying data in the Supporting Information file <Studieprotocol_ENG_versie2.pdf >. Due to the inclusion of these potentially identifying data, we have removed this file from your file inventory. Prior to sharing human research participant data, authors should consult with an ethics committee to ensure data are shared in accordance with participant consent and all applicable local laws.*

**Response:**

All identifying data have been removed and files anonymized.

**Changes in manuscript:**

- Supporting Information files

### **E15 – Supporting information captions**

*Please include captions for your Supporting Information files at the end of your manuscript, and update any in-text citations to match accordingly. Please see our Supporting Information guidelines for more information: http://journals.plos.org/plosone/s/supporting-information.*

**Response:**

Captions and in-text references have been added.

**Changes in manuscript:**

- End of manuscript (Lines 506-513)

# **REVIEWER #1** **R1.1 – ED trends and context**

*The reviewer notes that the decline in ED visits is likely a temporary post-COVID effect and that long-term ED use has followed demographic growth. They emphasise that ED crowding is a systemic issue driven by healthcare landscape changes, including fewer EDs and reduced hospital bed capacity. Additionally, the reviewer argues that the manuscript underrepresents the current capabilities of Dutch EDs and overstates that EDs are not designed to manage complex patients.*

**Response:**

We thank the reviewer for this insightful and important comment. We agree that the decline in ED visits observed during the COVID-19 period was temporary and that, in the longer term, ED utilisation has largely followed demographic trends. We have revised the Introduction accordingly to better reflect the role of demographic changes in driving ED demand.

We also agree that ED crowding is a complex, system‑level issue. Indeed, crowding is multifactorial, with input, throughput and output factors all contributing. We recognise that reduced ED availability and hospital bed capacity are important components of ED crowding, particularly because acute‑on‑chronic presentations often require complex diagnostic work‑ups and hospital admission. Although we did not explicitly detail these factors in the Introduction, we have revised the text to better emphasise the multifactorial nature of ED crowding. Given that our study focuses on acute‑on‑chronic presentations, we have placed greater emphasis on this aspect while acknowledging that other system‑level factors also play an important role.

Regarding the role and capabilities of modern emergency departments, we acknowledge that Dutch EDs have evolved into highly professionalised environments capable of managing a broad spectrum of acute conditions. We have revised the text to provide a more balanced and accurate description of current ED practice.

At the same time, we have clarified our wording to emphasise that, although EDs are well-equipped for acute diagnosis and stabilisation, they may not always be the best place to manage complex internal medicine patients, particularly those with multimorbidity or chronic care needs. We have adjusted the phrasing accordingly to avoid unintended underrepresentation of ED capabilities.

**Changes in manuscript:**

- Introduction (Lines 60–76)

### **R1.2 – Flashmob limitations**

*Study design: The authors have chosen for a flashmob-study, which comes with distinct limitations regarding scope, data, depth and bias. But I can understand the attractiveness of this method.*

**Response:**

We thank the reviewer for this clear and constructive appraisal of the flashmob study design.

We agree that this methodology provides a system-level observational snapshot and that its inherent characteristics limit temporal representativeness, seasonal variation, and causal inference. In line with the reviewer’s suggestion, we have strengthened the framing of the study design in the manuscript.

Specifically, we have clarified in the Methods section that this study represents a prospective, cross-sectional snapshot conducted over a single 24-hour period. In addition, we have expanded the Discussion to more explicitly emphasise that the findings should be interpreted as exploratory, descriptive estimates rather than definitive national prevalence measures. We also highlight the implications of the limited temporal window and the constraints for subgroup analyses and generalisability.

These additions aim to improve the methodological transparency and ensure appropriate interpretation of the results.

**Changes in manuscript:**

- Methods
  “This study is a prospective, cross-sectional, multicentre flashmob study conducted in the EDs of 28 Dutch hospitals, capturing a 24-hour observational snapshot of emergency department presentations.” (Lines 99-102)
- Discussion
  “In this 24-hour nationwide observational flashmob study, we evaluated the characteristics of internal medicine ED presentations across 28 hospitals, focusing on chronic disease related visits and the potential for redirection to alternative acute care settings. We found that nearly half of the ED visits were due to an acute complication of a chronic condition, highlighting the overlap between acute and chronic care. In 24% of these cases, the visit was considered preventable and in nearly 40%, patients could potentially have been redirected to alternative acute care settings, as retrospectively judged by the treating physician”.
  (Lines 264-270)
- Limitations
  “First, the findings represent descriptive estimates from a single 24‑hour observational snapshot and may therefore not fully capture temporal variation.”
  (Lines 336-338)

### **R1.3 – Specialty registration**

*Study setting: Not in every ED all patients are registered under a specific traditional specialty. The trend in the Netherlands since formal recognition of the specialty Emergency Medicine in January 2024 is towards registration also under emergency physicians names.*

**Response:**

We thank the reviewer for this clarification regarding registration practices in emergency departments in the Netherlands. We agree that, following the formal recognition of Emergency Medicine as a specialty in January 2024, there is an ongoing shift towards registration under emergency physicians.

To improve clarity and better reflect current practice, we have revised the manuscript accordingly. Specifically, we have replaced the phrasing with *“upon presentation or after clinical assessment”* to reflect that diagnostic categorisation (for DBC registration purposes) may occur either before or after clinical evaluation. As a dedicated DBC structure for emergency physicians is not yet routinely implemented, patients are often still registered under other specialties, depending on local practice.

**Changes in manuscript:**

- Methods
  “Upon presentation or after clinical assessment, patients are registered under a specific specialty (e.g., internal medicine) but may be assessed by an emergency physician (EP) or by a clinician from the corresponding specialty.” (Lines 110-113)

### **R1.4 – Referral misclassification**

*Study population: 'The study included all patients > 18 years presenting to the ED for internal medicine etc.' So prospective included, based upon referral. It is known that a significant part of referred patients end up having another problem then initially thought of and needing treatment of another specialism then referred to. In the Netherlands known to be around 20%. I'm curious how the authors dealt with this in their study.*

**Response:**

We thank the reviewer for this relevant observation. We agree that a proportion of patients initially referred to internal medicine may ultimately require management by another specialty, which reflects routine clinical practice in the Netherlands.

To clarify how this was addressed, we have revised the description of the study population in the manuscript in accordance with our study protocol. The study population comprises all patients aged ≥18 years who are seen in the emergency department for internal medicine, defined as patients who are referred to internal medicine, triaged to internal medicine, or for whom the internist ultimately acts as the primary treating physician.

This clarification ensures that both initial referral and final clinical responsibility are accounted for in the inclusion criteria.

**Changes in manuscript:**

- Methods
  “The study included all patients ≥18 years presenting to the ED for internal medicine (i.e. referred to internal medicine, triaged to internal medicine, or for whom the internist ultimately acted as the lead treating physician)”
  (Lines 116–120)

### **R1.5 – Retrospective assessment concerns**

*Study outcomes: '3) The proportion of ED visits that could have been redirected elsewhere in the emergency care chain or prevented, as retrospectively assessed by the treating physician.' First, Retrospectively assessed... The matter is that in practice it needs to be assessed prospectively. How does the retrospective assessment relate to ED/acute care practice? Second, by the treating physician only? On what grounds exactly? Can these be shared in the manuscript? How consistent were these assessments? Were these assessments reviewed by another physician? Can the physicians that actually did the assessments during the 24 hours perion be specified? Role? Experience?.*

**Response:**

We thank the reviewer for these important and constructive comments regarding the assessment of potentially redirectable ED visits.

First, we acknowledge that decisions regarding redirection in clinical practice are inherently prospective. In our study, however, a retrospective assessment was chosen as this allowed the treating physician to evaluate the full clinical course, including diagnostic results and outcomes, thereby enabling a more informed judgement on whether an ED visit might have been avoidable or suitable for alternative care pathways. We have clarified this rationale in the manuscript.

Second, the assessment was made by the treating physician, defined as the *primary responsible physician*, namely the consultant internist with final clinical responsibility for the patient. This approach ensured that the evaluation was performed by a clinician with full oversight of the patient’s presentation, diagnostic work-up, and clinical decision-making. We have further clarified this in the Methods section.

With regard to the grounds for assessment, we acknowledge that no predefined or standardised criteria were provided to guide these judgements. Physicians based their assessments on their clinical judgement and experience. We recognise that this may have introduced variability in how the possibility for redirection was interpreted, and this has now been explicitly addressed as a limitation in the manuscript.

Regarding consistency, we acknowledge that interobserver variability is a potential limitation of this approach. Given the pragmatic nature and exploratory intention of the flashmob study design, formal interrater reliability testing and secondary review were not performed. This limitation has now been explicitly stated in the manuscript and we added the advise to perform a prospective study to identify these possibilities in more detail.

**Changes in manuscript:**

- Methods – Study Outcomes

“The primary responsible physicians” (Lines 126-133)

- Methods – Data Collection
  “Second, patient level data were collected by the primary responsible physician at ED disposition through a separate eCRF, consisting of two sections (S2 Questionnaire 2). The first section captured clinical and demographic characteristics. The second section captured the primary responsible physicians assessment of the chronic disease related aspects of the presentation and the potential for alternative management. The primary responsible physician was asked to answer three key questions” (Lines 147-151)
- Results
  “Following clinical assessment, the primary responsible physicians were asked to retrospectively determine whether the ED presentation could have been prevented within the current organisation of care, or whether the patient could theoretically have been redirected to an alternative acute care setting, irrespective of existing care structures (Table 4).” (Lines 230-233)
- Discussion
  “This estimate should, however, be interpreted carefully, as the potential for redirection was assessed retrospectively after full ED evaluation. Nevertheless, the findings indicate that a substantial proportion of patients may be suitable for management in alternative acute care settings.” (Lines 293–295)
- Limitations
  “Furthermore, these assessments were made retrospectively by the primary responsible physician without predefined criteria, and interobserver variability was not assessed. This approach likely introduced insight, optimism, and post diagnostic reassessment bias, as physicians had access to full diagnostic information at the time of judgement.”
  (Lines 350-353).

### **R1.6 – Participation/refusal**

*Patient characteristics: How many patients have been asked to participate and how many refused?*

**Response:**

We thank the reviewer for this relevant question. Due to the pragmatic nature of the flashmob study design, it was not feasible to prospectively and systematically record the exact number of patients approached for participation or the number who declined at all participating sites.

To address this, we retrospectively requested inclusion data from participating hospitals. However, not all centres were able to provide these data, resulting in partial availability. For the hospitals that did report these figures (n=12), the majority of eligible patients were enrolled, with a median inclusion rate of 71.4% (range 44.4–100%).

Importantly, analyses comparing hospitals with lower inclusion rates to those with complete inclusion did not show differences in key outcomes, suggesting that the impact of selection bias is likely limited. Nevertheless, we acknowledge the incomplete availability of inclusion data as a limitation, which has been explicitly stated in the manuscript.

**In manuscript:**

- Results (Lines 258-262)
- Limitations (Lines 342-348)

### **R1.7 – Interpretation of redirectability**

*'In this prospective, nationwide flashmob study', Is the addition prospective appropriate? 'in nearly 40%, patients could theoretically have been redirected to alternative acute care settings as judged by the treating physician'. Based on the methodology used this is a too strong statement.*

**Response:**

We thank the reviewer for these insightful final comments.

First, regarding the term *“prospective”*, we agree that this may be open to interpretation in the context of a flashmob study design. As data were collected in real time during patient presentations, the study can be considered prospective in nature. However, to avoid potential ambiguity, we have clarified the wording in the manuscript.

Second, we agree that the original phrasing *“patients could theoretically have been redirected”* may be too strong given the retrospective and subjective nature of the assessment. We have therefore revised this statement to a more cautious formulation, emphasising that this reflects the judgement of the treating physician and should be interpreted accordingly.

**Changes in manuscript:**

- Results
  “Following clinical assessment, primary responsible physicians were asked to retrospectively determine whether the ED presentation could have been prevented within the current organisation of care, or whether the patient could theoretically have been redirected to an alternative acute care setting, irrespective of existing care structures (Table 4). In 49 cases (24.1%) the ED visit was considered preventable, and 81 patients (39.9%) were judged as eligible for redirection to alternative acute care settings. Among patients who presented with a complication of a chronic condition, 48.9% of ED visits were judged to have the potential to be redirected to an alternative setting”. (Lines 230-236)
- Discussion
  “This estimate should, however, be interpreted carefully, as the potential for redirection was assessed retrospectively after full ED evaluation. Nevertheless, the findings indicate that a substantial proportion of patients may be suitable for management in alternative acute care settings.” (Lines 293–295)
- Limitations
  “Furthermore, these assessments were made retrospectively by the primary responsible physician without predefined criteria, and interobserver variability was not assessed. This approach likely introduced insight, optimism, and post diagnostic reassessment bias, as physicians had access to full diagnostic information at the time of judgement.”
  (Lines 350-353).”

### **R1.9 – Broader system explanation**

*The authors spent a lot of attention on ‘acute on chronic care' for substantiation much of ED presentations could potentially be seen elsewhere in the hospital. But could they also reflect on what has made all this care being directed to the ED in the first place? What has caused, despite good alternatives (outpatient clinics, internal wards, acute medical units), that internal medicine patient flow has shifted to the EDs and away from the alternatives in the first place? Were there good reasons for that? How do the alternatives compare to the ED? I think this perspective might be of added value to the manuscript.?*

**Response:**

We thank the reviewer for this thoughtful suggestion. We agree that reflecting on the broader shift of internal medicine patient flow towards the ED is valuable. However, we consider a detailed analysis of this development to be beyond the scope of the present study, as it was not specifically investigated.

# **REVIEWER #2** **R2.1 – Flashmob design limitations**

*While some limitations are acknowledged, the Discussion should more explicitly emphasise that the findings represent exploratory, descriptive estimates rather than definitive national prevalence measures.*
*Explicitly framing the study as a system-level observational snapshot would strengthen methodological coherence and interpretation.*

**Response:**

We thank the reviewer for this clear and constructive appraisal of the flashmob study design.

We agree that this methodology provides an observational snapshot and that its inherent characteristics limit temporal representativeness, seasonal variation, and causal inference. In line with the reviewer’s suggestion, we have strengthened the framing of the study in the manuscript.

Specifically, we have clarified in the Methods section that this study represents a prospective, cross-sectional snapshot conducted over a single 24-hour period. In addition, we have expanded the Discussion to more explicitly emphasise that the findings should be interpreted as exploratory, descriptive estimates rather than definitive national prevalence measures. We also highlight the implications of the limited temporal window and the constraints for subgroup analyses and generalisability.

These additions aim to improve the methodological transparency and ensure appropriate interpretation of the results.

**Changes in manuscript:**

- Methods
  “This study is a prospective, cross-sectional, multicentre flashmob study conducted in the EDs of 28 Dutch hospitals, capturing a 24-hour observational snapshot of emergency department presentations.” (Lines 99-102)
- Limitations
  “First, the findings represent descriptive estimates from a single 24‑hour observational snapshot and may therefore not fully capture temporal variation.”
  (Lines 336-338)

### **R2.2 – Bias in outcomes**

*A key outcome—whether ED visits were “preventable” or “redirectable”—was determined retrospectively by the treating physician after ED evaluation.*
*This introduces:*
*• Hindsight bias*
*• Optimism bias*
*• Post-diagnostic reassessment bias*
*Because physicians had access to full diagnostic information at the time of judgement, these assessments may overestimate theoretical redirection feasibility.*
*Although acknowledged in the Limitations section, this issue should be more prominently integrated into the interpretation of the main results and conclusion..*

**Response:**

We thank the reviewer for this important and thoughtful comment. We agree that the retrospective assessment of preventability and redirection by the treating physician introduces potential sources of bias, including hindsight and post-diagnostic reassessment bias, as physicians had access to full clinical information at the time of judgement.

While this limitation was already acknowledged, we agree that it should be more prominently reflected in the interpretation of the findings. We have therefore revised the manuscript to more explicitly emphasise that these estimates represent theoretical and retrospective judgements, which may overestimate the feasibility of prevention and redirection in real-world, prospective decision-making.

At the same time, we would like to emphasise that this approach provides valuable insight into the potential scope for alternative care pathways. Although the exact proportions should be interpreted carefully, the findings consistently indicate that a substantial subgroup of patients may be suitable for care outside the ED, thereby highlighting an important area for further prospective research and organisational optimisation.

These considerations have now been more explicitly integrated into the Results and Discussion sections.

**Changes in manuscript:**

- Results
  “Following clinical assessment, primary responsible physicians were asked to retrospectively determine whether the ED presentation could have been prevented within the current organisation of care, or whether the patient could theoretically have been redirected to an alternative acute care setting, irrespective of existing care structures (Table 4).”(Lines 230-236)
- Discussion
  “This estimate should, however, be interpreted carefully, as the potential for redirection was assessed retrospectively after full ED evaluation. Nevertheless, the findings indicate that a substantial proportion of patients may be suitable for management in alternative acute care settings.” (Lines 293–295)
- Limitations
  “Furthermore, these assessments were made retrospectively by the primary responsible physician without predefined criteria, and interobserver variability was not assessed. This approach likely introduced insight, optimism, and post diagnostic reassessment bias, as physicians had access to full diagnostic information at the time of judgement. This may have resulted in an overestimation of potential redirection, as it is often challenging to assess a patient's condition and care needs prior to arrival at the ED.”
  (Lines 350-352).”

### **R2.3 – Generalisability**

*The study is conducted within the Dutch healthcare system, characterised by:*
*• Strong GP gatekeeping*
*• Structured referral systems*
*• Developed outpatient acute care infrastructure*
*These features significantly influence ED utilisation patterns.*
*The authors should more clearly discuss how these system characteristics may limit generalisability to:*
*• Open-access ED systems*
*• Healthcare environments with weaker primary care integration*
*• Different reimbursement models*
*Broader policy implications should therefore be framed cautiously and contextually.*

**Response:**

We thank the reviewer for this important comment regarding the generalisability of our findings. We agree that the Dutch healthcare system, characterised by strong GP gatekeeping, structured referral pathways, and the availability of outpatient acute care facilities, plays a key role in shaping ED utilisation patterns.

In response, we have expanded the Discussion to more explicitly reflect on how these system characteristics may influence the applicability of our findings to other healthcare settings. In particular, we emphasise that our results should be interpreted within the context of a healthcare system with strong primary care integration and referral structures. The extent to which similar patterns of preventable or redirectable ED visits are observed in settings with open-access ED systems or different organisational and reimbursement models may therefore vary.

We have incorporated this perspective in a dedicated paragraph in the Discussion and have framed the broader implications accordingly.

**Changes in manuscript:**

- Limitations
  Finally, the organisation of acute care within the Dutch healthcare systems may limit the generalisability of these findings to other healthcare settings with different referral structures or less gatekeeping and 24/7 access to GPs. However, given that demographic changes are occurring worldwide and ED crowding is a global challenge, our findings are still relevant internationally. (Lines 358–362)

### **R2.4 – Title clarity**

*While “FOCUS – Finding Optimal Care in Urgent Settings” is a memorable study acronym, the current title does not clearly convey the study population, setting, design, or main outcomes. In its present form, it provides limited information about the focus on internal medicine emergency department presentations, chronic disease–related visits, and the assessment of potential redirection to alternative acute care settings. I recommend revising the title to be more descriptive and informative, in line with PLOS ONE’s preference for titles that clearly reflect the content and scope of the study.*

**Response:**

We thank the reviewer for this helpful suggestion. We agree that the title should more clearly reflect the study population, design, and main outcomes. In line with this comment and PLOS ONE’s recommendations, we have revised the title to provide a more descriptive and informative summary of the study, while retaining the FOCUS acronym for clarity and recognition.

Chronic disease–related emergency department presentations and potential for redirection to alternative acute care settings (“FOCUS” study): a nationwide flashmob study.

**Changes in manuscript:**

- Title page

### **R2.5 – Language corrections**

*• In the Abstract (p. 10), “EDs are face increasing pressure” should be corrected to “EDs are facing increasing pressure.”*
*• Ensure consistent use of terminology throughout the manuscript (e.g., “acute on chronic,” “preventable,” “redirectable”).*

**Response:**

We thank the reviewer for these helpful observations. The grammatical error in the Abstract has been corrected to “EDs are facing increasing pressure.” In addition, we have carefully reviewed the manuscript to ensure consistent use of terminology, including terms such as “acute-on-chronic,” “preventable,” and “redirectable,” and have standardised these throughout the text.

**Changes in manuscript:**

- Entire manuscript

### **R2.6 – Tables readability**

*Tables 1–3 contain valuable information but are relatively dense. Consider condensing selected organisational details in Table 1 (p. 16) or relocating some elements to Supplementary Material to improve readability.*

**Response:**

We thank the reviewer for this helpful suggestion. In the previous revision, we already reduced the amount of detail presented in the tables by relocating selected information to the table notes and supplementary materials. We have carefully re-evaluated the tables and believe that they currently present the essential information in a concise manner, while preserving clarity and interpretability of the results.

Further reduction or relocation of content would risk omitting relevant context necessary for proper understanding of the data. For this reason, we have retained the current structure of the tables.
