# Supplementary material for: Chronic disease related emergency department presentations and potential for redirection to alternative acute care settings (“FOCUS” study): A nationwide flashmob study
Source: PLoS One. 2026 Jul 15;21(7):e0353157. doi: 10.1371/journal.pone.0353157 (PMC13372115; doi:10.1371/journal.pone.0353157)
Supplement: Attachment — Submitted filename: PONE-D-25-68026 2nd review.docx [file pone.0353157.s010.docx]

**Manuscript ID:** PONE-D-25-68026

Reviewer comments

I would like to thank the authors for their careful and thorough revision of the manuscript. The revised version shows clear improvement in several important aspects, particularly in the clarification of the title, correction of language in the abstract, and the inclusion of additional statements addressing methodological limitations.

The study remains timely and relevant, and the flashmob design continues to represent an innovative approach to capturing system-level characteristics of emergency care.

However, I believe that several points require further refinement, particularly regarding the interpretation of the findings. My remaining comments are as follows:

**1. Interpretation of Flashmob Design**

The authors have appropriately acknowledged that the study represents a 24-hour observational snapshot. However, the implications of this design should be more explicitly reflected in the interpretation of the findings.

The results should be consistently framed as **exploratory and descriptive estimates**, rather than as generalisable measures of prevalence or system performance.

**2. Subjectivity and Definition of Preventability and Redirection Outcomes**

The manuscript now acknowledges that preventability and redirection were assessed retrospectively by the treating physician.

However, a more fundamental methodological concern remains. These key outcomes appear to be based solely on the **subjective judgement of the primary responsible physician**, without predefined criteria, standardised definitions, or assessment of interobserver reliability. As currently presented, it is unclear how consistently these outcomes could be applied across different clinicians and settings.
