# Supplementary material for: Chronic disease related emergency department presentations and potential for redirection to alternative acute care settings (“FOCUS” study): A nationwide flashmob study
Source: PLoS One. 2026 Jul 15;21(7):e0353157. doi: 10.1371/journal.pone.0353157 (PMC13372115; doi:10.1371/journal.pone.0353157)
Supplement: Peer review history

# Peer review: Decision Letter 0

**DOI:** 10.1371/journal.pone.0353157.r001

**Authors:** Mabel Aoun

27 Feb 2026

PONE-D-25-68026

FOCUS - Finding Optimal Care in Urgent Settings

PLOS One

Dear Dr. Körnmann,

Thank you for submitting your manuscript to PLOS ONE. After careful consideration, we feel that it has merit but does not fully meet PLOS ONE’s publication criteria as it currently stands. Therefore, we invite you to submit a revised version of the manuscript that addresses the points raised during the review process.

==============================

**The manuscript was assessed by two reviewers and the academic editor. The research question is very relevant given the overcrowded emergency departments worldwide (not only in the Netherlands). However, a major revision of the manuscript is required. Please provide a point-by-point response to reviewers’ comments. The title should follow PLOS ONE requirements and include at the end the design of the study.  The reporting of the methods section needs some improvement. Please clarify in the study design that the study includes two steps, the first assessing patients and the second questioning doctors. Please provide the exact question asked to doctors. Please provide a clear paragraph of major variables collected with definitions especially for the variables included in the tables (what is U0, U1,…etc?).**

==============================

Please submit your revised manuscript by Apr 13 2026 11:59PM. If you will need more time than this to complete your revisions, please reply to this message or contact the journal office at plosone@plos.org. When you're ready to submit your revision, log on to https://www.editorialmanager.com/pone/ and select the 'Submissions Needing Revision' folder to locate your manuscript file.

Please include the following items when submitting your revised manuscript:

- A letter that responds to each point raised by the academic editor and reviewer(s). You should upload this letter as a separate file labeled 'Response to Reviewers'.
- A marked-up copy of your manuscript that highlights changes made to the original version. You should upload this as a separate file labeled 'Revised Manuscript with Track Changes'.
- An unmarked version of your revised paper without tracked changes. You should upload this as a separate file labeled 'Manuscript'.

If you would like to make changes to your financial disclosure, please include your updated statement in your cover letter. Guidelines for resubmitting your figure files are available below the reviewer comments at the end of this letter.

If applicable, we recommend that you deposit your laboratory protocols in protocols.io to enhance the reproducibility of your results. Protocols.io assigns your protocol its own identifier (DOI) so that it can be cited independently in the future. For instructions see: https://journals.plos.org/plosone/s/submission-guidelines#loc-laboratory-protocols. Additionally, PLOS ONE offers an option for publishing peer-reviewed Lab Protocol articles, which describe protocols hosted on protocols.io. Read more information on sharing protocols at https://plos.org/protocols?utm_medium=editorial-email&utm_source=authorletters&utm_campaign=protocols.

We look forward to receiving your revised manuscript.

Kind regards,

Mabel Aoun, MD, MPH

Academic Editor

PLOS One

Journal Requirements:

When submitting your revision, we need you to address these additional requirements.

1. Please ensure that your manuscript meets PLOS ONE's style requirements, including those for file naming. The PLOS ONE style templates can be found at

https://journals.plos.org/plosone/s/file?id=wjVg/PLOSOne_formatting_sample_main_body.pdf and https://journals.plos.org/plosone/s/file?id=ba62/PLOSOne_formatting_sample_title_authors_affiliations.pdf

2. Please include your full ethics statement in the ‘Methods’ section of your manuscript file. In your statement, please include the full name of the IRB or ethics committee who approved or waived your study, as well as whether or not you obtained informed written or verbal consent. If consent was waived for your study, please include this information in your statement as well.

3. One of the noted authors is a group or consortium [Acute Medicine Research Consortium (ORCA) and local investigators]. In addition to naming the author group, please list the individual authors and affiliations within this group in the acknowledgments section of your manuscript. Please also indicate clearly a lead author for this group along with a contact email address.

4. We note that you have indicated that there are restrictions to data sharing for this study. For studies involving human research participant data or other sensitive data, we encourage authors to share de-identified or anonymized data. However, when data cannot be publicly shared for ethical reasons, we allow authors to make their data sets available upon request. For information on unacceptable data access restrictions, please see http://journals.plos.org/plosone/s/data-availability#loc-unacceptable-data-access-restrictions.

Before we proceed with your manuscript, please address the following prompts:

a) If there are ethical or legal restrictions on sharing a de-identified data set, please explain them in detail (e.g., data contain potentially identifying or sensitive patient information, data are owned by a third-party organization, etc.) and who has imposed them (e.g., a Research Ethics Committee or Institutional Review Board, etc.). Please also provide contact information for a data access committee, ethics committee, or other institutional body to which data requests may be sent.

b) If there are no restrictions, please upload the minimal anonymized data set necessary to replicate your study findings to a stable, public repository and provide us with the relevant URLs, DOIs, or accession numbers. Please see http://www.bmj.com/content/340/bmj.c181.long for guidelines on how to de-identify and prepare clinical data for publication. For a list of recommended repositories, please see https://journals.plos.org/plosone/s/recommended-repositories. You also have the option of uploading the data as Supporting Information files, but we would recommend depositing data directly to a data repository if possible.

Please update your Data Availability statement in the submission form accordingly.

5. In the online submission form, you indicated that the data underlying this study contain potentially identifying and sensitive patient information and therefore cannot be publicly shared. This is particularly relevant given the study’s design as a 24-hour snapshot and the relatively small number of patients per hospital and medical specialty, which increases the risk of indirect identification of individual participants.

De-identified data may be made available upon reasonable request, subject to approval by the study steering committee and the relevant ethics committee, and in accordance with applicable data protection regulations. Data access requests should be addressed to the corresponding author.

De-identified data may be made available upon reasonable request, subject to approval by the study steering committee and the relevant ethics committee, and in accordance with applicable data protection regulations. Data access requests should be addressed to the corresponding author.

All PLOS journals now require all data underlying the findings described in their manuscript to be freely available to other researchers, either 1. In a public repository, 2. Within the manuscript itself, or 3. Uploaded as supplementary information.

This policy applies to all data except where public deposition would breach compliance with the protocol approved by your research ethics board. If your data cannot be made publicly available for ethical or legal reasons (e.g., public availability would compromise patient privacy), please explain your reasons on resubmission and your exemption request will be escalated for approval.

6. Thank you for stating the following financial disclosure:

This work was supported by a Science Grant of €7,500 from the Dutch Society of Acute Internal Medicine (Nederlandse Vereniging voor Internisten Acute Geneeskunde, NVIAG). No additional funding was received from public, commercial, or not-for-profit sectors.

Please state what role the funders took in the study. If the funders had no role, please state: "The funders had no role in study design, data collection and analysis, decision to publish, or preparation of the manuscript."

If this statement is not correct you must amend it as needed.

Please include this amended Role of Funder statement in your cover letter; we will change the online submission form on your behalf.

7. Please amend either the title on the online submission form (via Edit Submission) or the title in the manuscript so that they are identical.

8. Please amend your authorship list in your manuscript file to include author Michelle Noël Körnmann, Elisabeth Margaretha Mols, Harm R. Haak, Jelmer Alsma, Patricia Stassen, Karin Kaasjager, Geert Groeneveld, Marjolein N.T. Kremers.

9. Please amend the manuscript submission data (via Edit Submission) to include author E.M. Mols, M.N. Körnmann, H.R. Haak, J. Alsma, P. Stassen, H.A.H. Kaasjager, G.H. Groeneveld, M.N.T. Kremers.

10. One of the noted authors is a group or consortium [on behalf of the ORCA (Onderzoeks Consortium Acute Geneeskunde) Acute Medicine Research Consortium and local investigators].

In addition to naming the author group, please list the individual authors and affiliations within this group in the acknowledgments section of your manuscript. Please also indicate clearly a lead author for this group along with a contact email address.

11. We note that Figure 1 in your submission contain copyrighted images. All PLOS content is published under the Creative Commons Attribution License (CC BY 4.0), which means that the manuscript, images, and Supporting Information files will be freely available online, and any third party is permitted to access, download, copy, distribute, and use these materials in any way, even commercially, with proper attribution. For more information, see our copyright guidelines: http://journals.plos.org/plosone/s/licenses-and-copyright.

We require you to either (1) present written permission from the copyright holder to publish these figures specifically under the CC BY 4.0 license, or (2) remove the figures from your submission:

a. You may seek permission from the original copyright holder of Figure 1 to publish the content specifically under the CC BY 4.0 license.

We recommend that you contact the original copyright holder with the Content Permission Form (http://journals.plos.org/plosone/s/file?id=7c09/content-permission-form.pdf) and the following text:

“I request permission for the open-access journal PLOS ONE to publish XXX under the Creative Commons Attribution License (CCAL) CC BY 4.0 (http://creativecommons.org/licenses/by/4.0/). Please be aware that this license allows unrestricted use and distribution, even commercially, by third parties. Please reply and provide explicit written permission to publish XXX under a CC BY license and complete the attached form.”

Please upload the completed Content Permission Form or other proof of granted permissions as an "Other" file with your submission.

In the figure caption of the copyrighted figure, please include the following text: “Reprinted from [ref] under a CC BY license, with permission from [name of publisher], original copyright [original copyright year].”

b. If you are unable to obtain permission from the original copyright holder to publish these figures under the CC BY 4.0 license or if the copyright holder’s requirements are incompatible with the CC BY 4.0 license, please either i) remove the figure or ii) supply a replacement figure that complies with the CC BY 4.0 license. Please check copyright information on all replacement figures and update the figure caption with source information. If applicable, please specify in the figure caption text when a figure is similar but not identical to the original image and is therefore for illustrative purposes only.

12. We note you have included a table to which you do not refer in the text of your manuscript. Please ensure that you refer to Table 3 in your text; if accepted, production will need this reference to link the reader to the Table.

13. Please include a copy of Table 5 which you refer to in your text on page 12.

14. We note that there is identifying data in the Supporting Information file <Studieprotocol_ENG_versie2.pdf >. Due to the inclusion of these potentially identifying data, we have removed this file from your file inventory. Prior to sharing human research participant data, authors should consult with an ethics committee to ensure data are shared in accordance with participant consent and all applicable local laws.

Data sharing should never compromise participant privacy. It is therefore not appropriate to publicly share personally identifiable data on human research participants. The following are examples of data that should not be shared:

-Name, initials, physical address

-Ages more specific than whole numbers

-Internet protocol (IP) address

-Specific dates (birth dates, death dates, examination dates, etc.)

-Contact information such as phone number or email address

-Location data

-ID numbers that seem specific (long numbers, include initials, titled “Hospital ID”) rather than random (small numbers in numerical order)

Data that are not directly identifying may also be inappropriate to share, as in combination they can become identifying. For example, data collected from a small group of participants, vulnerable populations, or private groups should not be shared if they involve indirect identifiers (such as sex, ethnicity, location, etc.) that may risk the identification of study participants.

Additional guidance on preparing raw data for publication can be found in our Data Policy (https://journals.plos.org/plosone/s/data-availability#loc-human-research-participant-data-and-other-sensitive-data) and in the following article: http://www.bmj.com/content/340/bmj.c181.long.

Please remove or anonymize all personal information (<specific identifying information in file to be removed>), ensure that the data shared are in accordance with participant consent, and re-upload a fully anonymized data set. Please note that spreadsheet columns with personal information must be removed and not hidden as all hidden columns will appear in the published file.

15. Please include captions for your Supporting Information files at the end of your manuscript, and update any in-text citations to match accordingly. Please see our Supporting Information guidelines for more information: http://journals.plos.org/plosone/s/supporting-information.

16. If the reviewer comments include a recommendation to cite specific previously published works, please review and evaluate these publications to determine whether they are relevant and should be cited. There is no requirement to cite these works unless the editor has indicated otherwise.

[Note: HTML markup is below. Please do not edit.]

Reviewers' comments:

Reviewer's Responses to Questions

**Comments to the Author**

1. Is the manuscript technically sound, and do the data support the conclusions?

The manuscript must describe a technically sound piece of scientific research with data that supports the conclusions. Experiments must have been conducted rigorously, with appropriate controls, replication, and sample sizes. The conclusions must be drawn appropriately based on the data presented.

Reviewer #1: Partly

Reviewer #2: Yes

**********

2. Has the statistical analysis been performed appropriately and rigorously?

Reviewer #1: No

Reviewer #2: Yes

**********

3. Have the authors made all data underlying the findings in their manuscript fully available?

The PLOS Data policy requires authors to make all data underlying the findings described in their manuscript fully available without restriction, with rare exception (please refer to the Data Availability Statement in the manuscript PDF file). The data should be provided as part of the manuscript or its supporting information, or deposited to a public repository. For example, in addition to summary statistics, the data points behind means, medians and variance measures should be available. If there are restrictions on publicly sharing data—e.g. participant privacy or use of data from a third party—those must be specified.

Reviewer #1: Yes

Reviewer #2: Yes

**********

4. Is the manuscript presented in an intelligible fashion and written in standard English?

PLOS ONE does not copyedit accepted manuscripts, so the language in submitted articles must be clear, correct, and unambiguous. Any typographical or grammatical errors should be corrected at revision, so please note any specific errors here.

Reviewer #1: Yes

Reviewer #2: Yes

**********

5. Review Comments to the Author

Please use the space provided to explain your answers to the questions above. You may also include additional comments for the author, including concerns about dual publication, research ethics, or publication ethics. (Please upload your review as an attachment if it exceeds 20,000 characters)

Reviewer #1: Many thanks to the authors for investigating possibilities to relieve pressure from Dutch emergency departments (EDs) by assessing the potential for redirection to alternative acute care settings, providing a foundation fo optimizing the delivery of care in the right place at the right time. Nobody will oppose to that. However, reading the manuscript I have the following remarks and concerns:

INTRODUCTION PART

The authors state a decline in ED visits in recent years. Indeed this is known as a temporary postcovid effect. However, since 2000 the growth of ED-visits is reasonable in line with demographic growth. The post-corona dip has now been overtaken and the pre-corona trend restored.

A more plausible explanation may indeed lie in the increasing complexity of patient care needs, driven by an aging population with multiple health problems.

However, when it comes to pressure on acute care and crowded EDs the authors do not mention anything about landscape changes that have taken place over the last 15 years and played an major role in the rising number of overcrowded EDs and ED diversions. A steady growing total number of ED-visits over the years, with 25% less EDs and an enormous reduction of hospital beds behind the remaining EDs overthe last 15 years. The ED landscape has stiffened.

ED crowding is a complex and systemic problem, expressing itself in the ED. The complexity ED-crowding, if mentioned, should be brought out better.

The authors state that: Traditionally, EDs are not designed to provide care to patients with complex needs, due to their primary focus on trauma care and diagnosis-driven pathways. However, a shift in the acute care landscape had occured, with an increasing number of patients presenting to the ED due to detoriation or complications of pre-existing chronic illnesses.'

True, but 25 - 20 years ago. Since then EDs in the Netherlands have developed long ago to areas of a medical facilities is not adequate for certain devoted to the provision of an organized system of emergency medical care that is staffed by Medical Specialist Physicians and/or Emergency Physicians and has the basis resources to resuscitate, diagnose and treat patients with medical emergencies of any kind (illness, trauma and acute mental issues). The authors paint too modest a picture of the highly and broad professionalized current EDs in the Netherlands, suggesting ED-design is a problem to handle certain presentations. This is not substantiated.

Having made these comments, ofcourse it is good to see whether internal medicine problems can be seen outside the ED safely, cost effective and with enough personel (the biggest tightness).

METHODS

Study design: The authors have chosen for a flashmob-study, which comes with distict limitations regarding scope, data, depth and bias. But I can understand the attractiveness of this method.

Study setting: Not in every ED all patients are registered under a specific traditional specialty. The trend in the Netherlands since formal recognition of the specialty Emergency Medicine in January 2024 is towards registration also under emergency physicians names.

Study population: 'The study included all patients > 18 years presenting t the ED for internal medicine etc.' So prospective included, based upon referral. It is known that a significant part of referred patients end up having another problem then initially thought of and needing treatment of another specialism then referred to. In the Netherlands known to be around 20%. I'm curious how the authors dealt with this in their study.

Study outcomes: '3) The proportion of ED visits that could have been redirected elsewhere in the emergency care chain or prevented, as retrospectively assessed by the treating physician.' First, Retrospectively assessed... The matter is that in practice it needs to be assessed prospectively. How does the retrospective assessment relate to ED/acute care practice? Second, by the treating physician only? On what grounds exactly? Can these be shared in the manuscript? How consistent were these assessments? Were these assessments reviewed by another physician? Can the physicians that actually did the assessments during the 24 hours perion be specified? Role? Experience?

RESULTS

Patient chracteristics: How many patients have been asked to participate and how many refused?

Redirecting urgent care: I reiterate my concerns about the methodology used and as a result these Results.

DISCUSSION

'In this prospective, nationwide flashmob study', Is the addition prospective appropriate?

'in nearly 40%, patients could theoretically have been redirected to alternative acute care settings as judged by the treating physician'. Based on the methodology used this is a too strong statement.

The authors spent a lot of attention on ácute on chronic care' for substantiation much of ED presentations could potentially be seen elsewhere in the hospital. But could they also reflect on what has made all this care being directed to the ED in the first place? What has caused, despite good alternatives (out patient clinics, internal wards, acute medical units), that internal medicne patient flow has shifted to the EDs and away from the alternatives in the first place? Were there good reasons for that? How do the alternatives compare to the ED? I think this perspective might be of added value to the manuscript.

Reviewer #2: Overall Assessment

This manuscript presents a nationwide, prospective multicentre flashmob study conducted across 28 Dutch hospitals, evaluating the proportion of internal medicine emergency department (ED) visits related to chronic conditions and the potential for redirection to alternative acute care settings.

The research question is timely and highly relevant in the context of ED crowding, workforce constraints, and the increasing burden of chronic disease. The flashmob methodology—characterised by short-term, simultaneous, prospective data collection across multiple centres—is innovative and appropriate for capturing a real-time snapshot of acute care delivery at the system level.

Major Comments

1. Flashmob Methodology: Appropriateness and Inherent Constraints

The use of a flashmob design is a central methodological feature of this study (Methods section).

This approach is appropriate for descriptive, system-level questions and allows broad national participation with limited resources. However, the snapshot nature of flashmob studies inherently limits:

• Temporal representativeness (single 24-hour window)

• Seasonal variability

• Weekday vs weekend variation

• Causal inference

• Precision of subgroup analyses

While some limitations are acknowledged, the Discussion should more explicitly emphasise that the findings represent exploratory, descriptive estimates rather than definitive national prevalence measures.

Explicitly framing the study as a system-level observational snapshot would strengthen methodological coherence and interpretation.

2. Subjectivity of Key Outcomes (Preventability and Redirection)

A key outcome—whether ED visits were “preventable” or “redirectable”—was determined retrospectively by the treating physician after ED evaluation.

This introduces:

• Hindsight bias

• Optimism bias

• Post-diagnostic reassessment bias

Because physicians had access to full diagnostic information at the time of judgement, these assessments may overestimate theoretical redirection feasibility.

Although acknowledged in the Limitations section, this issue should be more prominently integrated into the interpretation of the main results and conclusion.

3. External Generalisability

The study is conducted within the Dutch healthcare system, characterised by:

• Strong GP gatekeeping

• Structured referral systems

• Developed outpatient acute care infrastructure

These features significantly influence ED utilisation patterns.

The authors should more clearly discuss how these system characteristics may limit generalisability to:

• Open-access ED systems

• Healthcare environments with weaker primary care integration

• Different reimbursement models

Broader policy implications should therefore be framed cautiously and contextually.

Minor Comments

1. Comment on the Title

While “FOCUS – Finding Optimal Care in Urgent Settings” is a memorable study acronym, the current title does not clearly convey the study population, setting, design, or main outcomes. In its present form, it provides limited information about the focus on internal medicine emergency department presentations, chronic disease–related visits, and the assessment of potential redirection to alternative acute care settings. I recommend revising the title to be more descriptive and informative, in line with PLOS ONE’s preference for titles that clearly reflect the content and scope of the study.

2. Abstract and Language

• In the Abstract (p. 10), “EDs are face increasing pressure” should be corrected to “EDs are facing increasing pressure.”

• Ensure consistent use of terminology throughout the manuscript (e.g., “acute on chronic,” “preventable,” “redirectable”).

3. Tables

• Tables 1–3 contain valuable information but are relatively dense. Consider condensing selected organisational details in Table 1 (p. 16) or relocating some elements to Supplementary Material to improve readability.

**********

6. PLOS authors have the option to publish the peer review history of their article (what does this mean?). If published, this will include your full peer review and any attached files.

If you choose “no”, your identity will remain anonymous but your review may still be made public.

**Do you want your identity to be public for this peer review?**  For information about this choice, including consent withdrawal, please see our Privacy Policy.

Reviewer #1: No

Reviewer #2: No

**********

[NOTE: If reviewer comments were submitted as an attachment file, they will be attached to this email and accessible via the submission site. Please log into your account, locate the manuscript record, and check for the action link "View Attachments". If this link does not appear, there are no attachment files.]

To ensure your figures meet our technical requirements, please review our figure guidelines: https://journals.plos.org/plosone/s/figures

You may also use PLOS’s free figure tool, NAAS, to help you prepare publication quality figures: https://journals.plos.org/plosone/s/figures#loc-tools-for-figure-preparation.

NAAS will assess whether your figures meet our technical requirements by comparing each figure against our figure specifications.

# Author comment: Author response to Decision Letter 1

**DOI:** 10.1371/journal.pone.0353157.r002

11 Apr 2026

Response to reviewers

Dear editor and reviewers,

We would like to sincerely thank you and the reviewers for your careful evaluation of our manuscript and for the constructive and insightful comments provided. We greatly appreciate the time and effort invested in reviewing our work. The feedback has helped us to significantly improve the clarity, transparency, and overall quality of the manuscript.

We have revised the manuscript thoroughly in accordance with all editorial and reviewer comments. We provided a structured summary of the major revisions made in the response to the reviewers letter.

We believe that these revisions have substantially strengthened the manuscript and addressed all concerns raised. We hope that the revised version meets the journals standards for publication.

Thank you again for your consideration.

Sincerely,

M.N. Körnmann & E.M. Mols

Attachment Submitted filename: FOCUS Response to Reviewers.docx

# Peer review: Decision Letter 1

**DOI:** 10.1371/journal.pone.0353157.r003

**Authors:** Mabel Aoun, Vijayaprakash Suppiah

8 Jun 2026

PONE-D-25-68026R1

Chronic disease related emergency department presentations and potential for redirection to alternative acute care settings (“FOCUS” study): a nationwide flashmob study

PLOS One

Dear Dr. Körnmann,

Thank you for submitting your manuscript to PLOS ONE. After careful consideration, we feel that it has merit but does not fully meet PLOS ONE’s publication criteria as it currently stands. Therefore, we invite you to submit a revised version of the manuscript that addresses the points raised during the review process.

Please submit your revised manuscript by Jul 23 2026 11:59PM. If you will need more time than this to complete your revisions, please reply to this message or contact the journal office at plosone@plos.org. When you're ready to submit your revision, log on to https://www.editorialmanager.com/pone/ and select the 'Submissions Needing Revision' folder to locate your manuscript file.

Please include the following items when submitting your revised manuscript:

- A letter that responds to each point raised by the academic editor and reviewer(s). You should upload this letter as a separate file labeled 'Response to Reviewers'.
- A marked-up copy of your manuscript that highlights changes made to the original version. You should upload this as a separate file labeled 'Revised Manuscript with Track Changes'.
- An unmarked version of your revised paper without tracked changes. You should upload this as a separate file labeled 'Manuscript'.

If you would like to make changes to your financial disclosure, please include your updated statement in your cover letter. Guidelines for resubmitting your figure files are available below the reviewer comments at the end of this letter.

If applicable, we recommend that you deposit your laboratory protocols in protocols.io to enhance the reproducibility of your results. Protocols.io assigns your protocol its own identifier (DOI) so that it can be cited independently in the future. For instructions see: https://journals.plos.org/plosone/s/submission-guidelines#loc-laboratory-protocols. Additionally, PLOS ONE offers an option for publishing peer-reviewed Lab Protocol articles, which describe protocols hosted on protocols.io. Read more information on sharing protocols at https://plos.org/protocols?utm_medium=editorial-email&utm_source=authorletters&utm_campaign=protocols.

As the corresponding author, your ORCID iD is verified in the submission system and will appear in the published article. PLOS supports the use of ORCID, and we encourage all coauthors to register for an ORCID iD and use it as well. Please encourage your coauthors to verify their ORCID iD within the submission system before final acceptance, as unverified ORCID iDs will not appear in the published article. *Only*  the individual author can complete the verification step; PLOS staff *cannot*  verify ORCID iDs on behalf of authors.

We look forward to receiving your revised manuscript.

Kind regards,

Vijayaprakash Suppiah, PhD

Academic Editor

PLOS One

Journal Requirements:

1. If the reviewer comments include a recommendation to cite specific previously published works, please review and evaluate these publications to determine whether they are relevant and should be cited. There is no requirement to cite these works unless the editor has indicated otherwise.

2. Please review your reference list to ensure that it is complete and correct. If you have cited papers that have been retracted, please include the rationale for doing so in the manuscript text, or remove these references and replace them with relevant current references. Any changes to the reference list should be mentioned in the rebuttal letter that accompanies your revised manuscript. If you need to cite a retracted article, indicate the article’s retracted status in the References list and also include a citation and full reference for the retraction notice.

[Note: HTML markup is below. Please do not edit.]

Reviewer's Responses to Questions

**Comments to the Author**

1. If the authors have adequately addressed your comments raised in a previous round of review and you feel that this manuscript is now acceptable for publication, you may indicate that here to bypass the “Comments to the Author” section, enter your conflict of interest statement in the “Confidential to Editor” section, and submit your "Accept" recommendation.

Reviewer #2: (No Response)

**********

2. Is the manuscript technically sound, and do the data support the conclusions?

The manuscript must describe a technically sound piece of scientific research with data that supports the conclusions. Experiments must have been conducted rigorously, with appropriate controls, replication, and sample sizes. The conclusions must be drawn appropriately based on the data presented.

Reviewer #2: (No Response)

**********

3. Has the statistical analysis been performed appropriately and rigorously?

Reviewer #2: (No Response)

**********

4. Have the authors made all data underlying the findings in their manuscript fully available?

The PLOS Data policy requires authors to make all data underlying the findings described in their manuscript fully available without restriction, with rare exception (please refer to the Data Availability Statement in the manuscript PDF file). The data should be provided as part of the manuscript or its supporting information, or deposited to a public repository. For example, in addition to summary statistics, the data points behind means, medians and variance measures should be available. If there are restrictions on publicly sharing data—e.g. participant privacy or use of data from a third party—those must be specified.

Reviewer #2: (No Response)

**********

5. Is the manuscript presented in an intelligible fashion and written in standard English?

PLOS ONE does not copyedit accepted manuscripts, so the language in submitted articles must be clear, correct, and unambiguous. Any typographical or grammatical errors should be corrected at revision, so please note any specific errors here.

Reviewer #2: (No Response)

**********

6. Review Comments to the Author

Please use the space provided to explain your answers to the questions above. You may also include additional comments for the author, including concerns about dual publication, research ethics, or publication ethics. (Please upload your review as an attachment if it exceeds 20,000 characters)

Reviewer #2: (No Response)

**********

7. PLOS authors have the option to publish the peer review history of their article (what does this mean?). If published, this will include your full peer review and any attached files.

If you choose “no”, your identity will remain anonymous but your review may still be made public.

**Do you want your identity to be public for this peer review?**  For information about this choice, including consent withdrawal, please see our Privacy Policy.

Reviewer #2: No

**********

[NOTE: If reviewer comments were submitted as an attachment file, they will be attached to this email and accessible via the submission site. Please log into your account, locate the manuscript record, and check for the action link "View Attachments". If this link does not appear, there are no attachment files.]

To ensure your figures meet our technical requirements, please review our figure guidelines: https://journals.plos.org/plosone/s/figures

You may also use PLOS’s free figure tool, NAAS, to help you prepare publication quality figures: https://journals.plos.org/plosone/s/figures#loc-tools-for-figure-preparation.

NAAS will assess whether your figures meet our technical requirements by comparing each figure against our figure specifications.

Attachment Submitted filename: PONE-D-25-68026 2nd review.docx

# Author comment: Author response to Decision Letter 2

**DOI:** 10.1371/journal.pone.0353157.r004

18 Jun 2026

Dear Editor and Reviewers,

We would like to thank you for your careful evaluation of the revised manuscript and for your additional constructive comments. We appreciate the opportunity to further improve our work.

We have revised the manuscript accordingly and have addressed all remaining comments. A detailed point-by-point response is provided in the Response to Reviewers document.

We hope that the revised manuscript satisfactorily addresses the reviewers’ concerns and is now suitable for publication.

Thank you again for your consideration.

Sincerely,

M.N. Körnmann & E.M. Mols

On behalf of all authors

m.n.koernmann@amsterdamumc.nl

Attachment Submitted filename: FOCUS_Response_to_Reviewers_auresp_2.docx

# Editor report: Decision Letter 2

**DOI:** 10.1371/journal.pone.0353157.r005

**Authors:** Mabel Aoun, Vijayaprakash Suppiah, Vijayaprakash Suppiah

21 Jun 2026

Chronic disease related emergency department presentations and potential for redirection to alternative acute care settings (“FOCUS” study): a nationwide flashmob study

PONE-D-25-68026R2

Dear Dr. Körnmann,

We’re pleased to inform you that your manuscript has been judged scientifically suitable for publication and will be formally accepted for publication once it meets all outstanding technical requirements.

Within one week, you’ll receive an e-mail detailing the required amendments. When these have been addressed, you’ll receive a formal acceptance letter and your manuscript will be scheduled for publication.

An invoice will be generated when your article is formally accepted. Please note, if your institution has a publishing partnership with PLOS and your article meets the relevant criteria, all or part of your publication costs will be covered. Please make sure your user information is up-to-date by logging into Editorial Manager at Editorial Manager® and clicking the ‘Update My Information' link at the top of the page. For questions related to billing, please contact billing support.

If your institution or institutions have a press office, please notify them about your upcoming paper to help maximize its impact. If they’ll be preparing press materials, please inform our press team as soon as possible -- no later than 48 hours after receiving the formal acceptance. Your manuscript will remain under strict press embargo until 2 pm Eastern Time on the date of publication. For more information, please contact onepress@plos.org.

Kind regards,

Vijayaprakash Suppiah, PhD

Academic Editor

PLOS One

# Editor report: Acceptance letter

**DOI:** 10.1371/journal.pone.0353157.r006

**Authors:** Mabel Aoun, Vijayaprakash Suppiah, Vijayaprakash Suppiah

PONE-D-25-68026R2

PLOS One

Dear Dr. Körnmann,

I'm pleased to inform you that your manuscript has been deemed suitable for publication in PLOS One. Congratulations! Your manuscript is now being handed over to our production team.

At this stage, our production department will prepare your paper for publication. This includes ensuring the following:

* All references, tables, and figures are properly cited

* All relevant supporting information is included in the manuscript submission,

* There are no issues that prevent the paper from being properly typeset

You will receive further instructions from the production team, including instructions on how to review your proof when it is ready. Please keep in mind that we are working through a large volume of accepted articles, so please give us a few days to review your paper and let you know the next and final steps.

Lastly, if your institution or institutions have a press office, please let them know about your upcoming paper now to help maximize its impact. If they'll be preparing press materials, please inform our press team within the next 48 hours. Your manuscript will remain under strict press embargo until 2 pm Eastern Time on the date of publication. For more information, please contact onepress@plos.org.

You will receive an invoice from PLOS for your publication fee after your manuscript has reached the completed accept phase. If you receive an email requesting payment before acceptance or for any other service, this may be a phishing scheme. Learn how to identify phishing emails and protect your accounts at https://explore.plos.org/phishing.

If we can help with anything else, please email us at customercare@plos.org.

Thank you for submitting your work to PLOS ONE and supporting open access.

Kind regards,

PLOS ONE Editorial Office Staff

on behalf of

Dr. Vijayaprakash Suppiah

Academic Editor

PLOS One
